# Recording of cellular physiological histories along optically readable self-assembling protein chains

**DOI:** 10.1038/s41587-022-01586-7

**Published:** 2023-01-02

**Authors:** Changyang Linghu, Bobae An, Monika Shpokayte, Orhan T. Celiker, Nava Shmoel, Ruihan Zhang, Chi Zhang, Demian Park, Won Min Park, Steve Ramirez, Edward S. Boyden

**Affiliations:** 1 Brain and Cognitive Sciences, MIT, Cambridge, MA USA; 2 Biological Engineering, MIT, Cambridge, MA USA; 3 Media Arts and Sciences, MIT, Cambridge, MA USA; 4 McGovern Institute, MIT, Cambridge, MA USA; 5 Graduate Program for Neuroscience, Boston University, Boston, MA USA; 6 Psychological and Brain Sciences, Boston University, Boston, MA USA; 7 Chemical Engineering, Kansas State University, Manhattan, KS USA; 8 Howard Hughes Medical Institute, MIT, Cambridge, MA USA; 9 K Lisa Yang Center for Bionics, MIT, Cambridge, MA USA; 10 Center for Neurobiological Engineering, MIT, Cambridge, MA USA; 11 Koch Institute, MIT, Cambridge, MA USA; 12 Present Address: Department of Cell and Developmental Biology, Program in Single Cell Spatial Analysis, and Michigan Neuroscience Institute, University of Michigan Medical School, Ann Arbor, MI USA

**Keywords:** Synthetic biology, Protein design, Reporter genes, Optical imaging

## Abstract

Observing cellular physiological histories is key to understanding normal and disease-related processes. Here we describe expression recording islands—a fully genetically encoded approach that enables both continual digital recording of biological information within cells and subsequent high-throughput readout in fixed cells. The information is stored in growing intracellular protein chains made of self-assembling subunits, human-designed filament-forming proteins bearing different epitope tags that each correspond to a different cellular state or function (for example, gene expression downstream of neural activity or pharmacological exposure), allowing the physiological history to be read out along the ordered subunits of protein chains with conventional optical microscopy. We use expression recording islands to record gene expression timecourse downstream of specific pharmacological and physiological stimuli in cultured neurons and in living mouse brain, with a time resolution of a fraction of a day, over periods of days to weeks.

## Abstract (web summary)

A history of cellular events is recorded in self-assembling protein chains.

## Main

Reading out biological signals and processes that take place over time in living cells, organs and organisms is essential to advancing basic and translational biological research. The imaging of genetically encoded fluorescent signal reporters, for example, enables specific biological activities to be monitored in real time in living cells^1^. However, long-term live imaging is laborious and equipment intensive, because a single microscope often has to be monopolized for the duration of the experiment. Furthermore, the number of cells that can be observed is limited by the performance of live imaging methods, which are not as scalable as fixed-tissue imaging methods. The latter benefit from sectioning, clearing, expansion and other techniques that improve the number of cells that can be surveyed, the resolution and the number of signals that can be analyzed at once^2–4^. Snapshot methods such as RNA fluorescence in situ hybridization (FISH)^5^ and protein immunostaining^6^ can enable one (and sometimes two) time points of a physiological signal to be inferred in fixed cells, but cannot support continuous recording of physiological signals for later fixed-cell readout. Nevertheless, these methods allow biological information readout over very large spatial scales, even entire mammalian brains, because fixed cells or tissues can be scalably imaged thanks to postpreservation tissue processing.

**Fig. 1:**
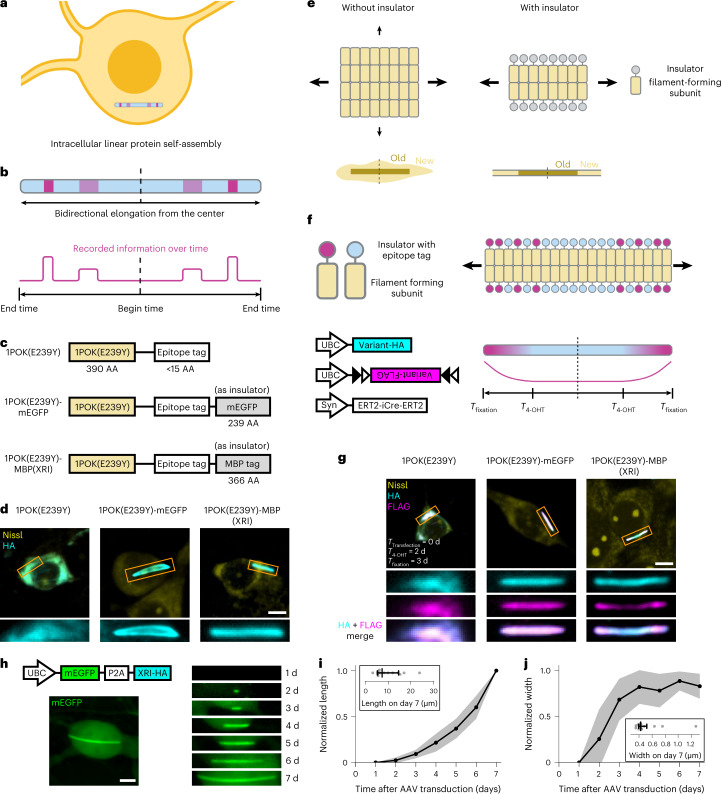
Concept and development of linear protein self-assembly-based cellular physiology recording devices. **a**, Schematic of intracellular linear protein self-assembly. **b**, Bidirectional elongating intracellular linear protein self-assembly for encoding, storing and reading out biological information. Blue shading, components on the self-assembly whose expression is constitutive over time; red shading, components on the self-assembly whose expression is dependent on biological events of interest over time; red line, density along the self-assembly of the components whose expression is dependent on biological events of interest over time. **c**, Schematic of variants of self-assembling proteins. XRI, the variant selected as the XRI design throughout this paper; AA, amino acid. **d**, Representative confocal images of cultured mouse hippocampal neurons expressing self-assembling protein variants with the epitope tag HA, taken after fixation, Nissl staining and immunostaining against the HA tag. Scale bar, 5 µm throughout this figure. Rectangular panels at the bottom, enlarged views of regions marked in orange rectangles in the top row of square panels. **e**, Schematic of protein self-assemblies without (left) and with (right) an insulator component fused to each of the filament-forming subunits. Arrows with different sizes, growth directions of protein self-assemblies, with arrow sizes indicating growth rates; old, subunits that bound to the protein self-assembly earlier; new, subunits that are binding to the protein self-assembly currently. **f**, Schematic of the protein self-assembly and the constructs in the chemically induced gene expression experiment. Variant-HA, self-assembling protein variant (1POK(E239Y), 1POK(E239Y)-mEGFP or 1POK(E239Y)-MBP) with the epitope tag HA; Variant-FLAG, self-assembling protein variant with the epitope tag FLAG; Syn, human synapsin promoter; black and white triangles, lox sites in the FLEX construct; *T*_4-OHT_, the time at which cells are treated with 4-OHT; *T*_fixation_, the time at which cells are fixed. **g**, Representative confocal images of cultured mouse hippocampal neurons expressing constructs (bottom left of **f**), taken after fixation, Nissl staining and immunostaining against the HA tag and the FLAG tag. *T*_transfection_, the time at which the constructs are delivered to cells via DNA transfection. Three rows of rectangular panels at the bottom, enlarged views of regions marked in orange rectangles in the top row of square panels. **h**, Representative confocal images of a live cultured mouse hippocampal neuron expressing mEGFP-P2A-XRI-HA. Top left, construct schematic; bottom left, image taken 7 days after AAV transduction under the GFP channel; right, images of the XRI protein self-assembly in the same neuron as in the bottom left taken 1–7 days after AAV transduction, showing the GFP channel. **i**,**j**, Normalized length (**i**) and width (**j**) of XRI versus time after AAV transduction (*n* = 14 XRIs from eight neurons from one culture; length and width were normalized to the maximum values over time, respectively). Centerline, mean; shaded boundary, s.d.; insets, absolute length and width of XRI at 7 days after AAV transduction; middle vertical line, median; error bar, interquartile range; gray dots, individual datapoints.

If biological information could be recorded by cells and stored digitally within their cellular volumes for later readout after cell fixation, it would be possible to combine both recording of continuous time histories of physiological signals and scalable signal history readout. Several studies have demonstrated the recording of cellular histories in nucleic acids, for readout through sequencing after cells or tissues are dissociated and/or lysed^7–20^. However, one often wants to study intact cells, tissues or organs. Here, we achieve this by recording biological information along growing protein chains made of fully genetically encoded self-assembling proteins, which bear different labels that encode different cellular states or functions. While the cell is alive, the self-assembling, label-bearing proteins are added constantly to the growing chain, enabling continuous recording of the presence of the different label-bearing proteins that are available (Fig. 1a,b). For example, if, at a certain point in time, proteins with one label are common, and proteins with another label are rare, the part of the chain that is growing at the current moment in time will acquire more of the former label than the latter. In other words, the local density of labels will favor the first label over the second, even if the labels are independent and being added at a constant rate. When the experiment is done, the chain of proteins can be read out by ordinary immunostaining and imaging after cell or tissue fixation.

We show that this expression recording island (XRI) strategy can be used for long-term recording of gene expression timecourse, with single-cell precision, across cell populations, without altering normal cell physiology or cell health. Because the linear protein assembly grows continuously over time, it acts like a molecular tape recorder that preserves the temporal order of the protein monomers made available by the cell depending on the current state or function of the cell. For example, if protein monomers with the epitope tag ‘A’ are steadily expressed by the cell, and the expression of protein monomers with the epitope tag ‘B’ is increased by, for example, a neural-activity-dependent promoter, then the neural-activity-dependent event will result in permanent storage of the activity record in the order of the epitope tags along the growing protein chain, enabling later readout via immunostaining against tags ‘A’ and ‘B’ followed by standard imaging. We applied XRIs to perform 4-day recordings of, amongst other things, c-fos-promoter-driven gene expression in cultured mouse hippocampal neurons after depolarization, and also show that pharmacological modulation of gene expression histories in the living mouse brain could also be read out post hoc.

## Results

**Extended Data Fig. 1:**
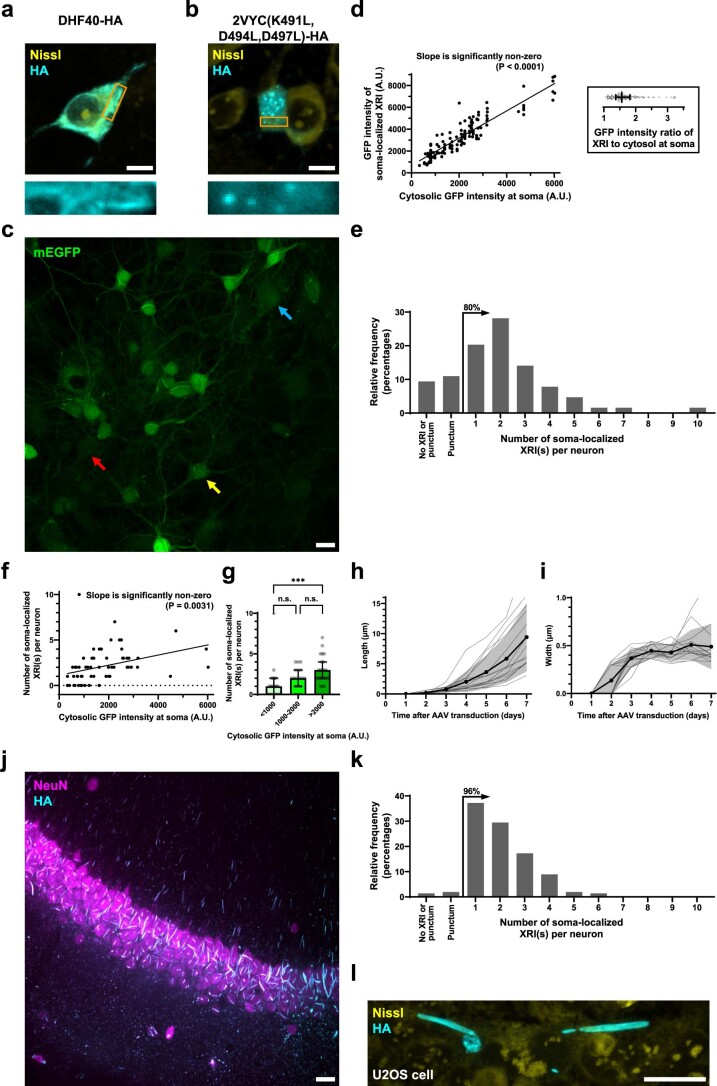
Analysis of XRI formation in neuron cultures and in mouse brains. (**a-b**) Representative confocal images of cultured mouse hippocampal neurons expressing (**a**) DHF40 or (**b**) 2VYC(K491L,D494L,D497L) with epitope tag HA, taken after fixation, Nissl staining, and immunostaining against the HA tag. Scale bars, 5 µm. Rectangular panels at the bottom, enlarged views of regions marked in orange rectangles in the top row of square panels. See Supplementary Table 1 for sequences of the motifs; see Supplementary Table 2 for all tested constructs. (**c**) A representative confocal image in the GFP channel of cultured mouse hippocampal neurons 7 days after AAV transduction of AAV9-UBC-mEGFP-P2A-XRI-HA. Bicistronic co-expression of mEGFP with XRI (via P2A) allows estimations of the level of AAV delivery in individual cells using GFP intensity in the cytosol. Colored arrows indicate representative neurons with fiber-like structures (that is, successful formation of XRI assemblies; yellow arrow), punctum-like structures (blue arrow), or no resolvable structure (red arrow). Scale bar, 20 µm. (**d**) Left, scatter plot of the GFP intensity of soma-localized XRI versus cytosolic GFP intensity (black line, line fit from linear regression; P value, two-sided F test with the null hypothesis that the slope is zero); right, the ratio of the GFP intensity of soma-localized XRI to the cytosolic GFP intensity (middle vertical line, median; error bar, interquartile range; gray dots, individual data points); n = 134 XRIs from 51 neurons with soma-localized XRIs from 4 fields of view from 1 culture. Throughout this figure: cytosolic GFP intensities were measured by averaging the pixel intensity values across pixels within the soma but outside the XRIs, from images captured under the same imaging condition in the GFP channel; XRI GFP intensities were measured by averaging the pixel intensity values along individual XRIs in these images. (**e**) Histogram of the number of soma-localized XRIs per neuron, among GFP-positive neurons (n = 64 neurons from 4 fields of view from 1 culture), 7 days after AAV transduction of AAV9-UBC-mEGFP-P2A-XRI-HA. ‘80%’ with an arrow, 80% of the neurons had soma-localized XRI(s). (**f**) Scatter plot of the number of soma-localized XRIs per neuron versus the cytosolic GFP intensity for neurons in **e**. Black line, line fit from linear regression; P value, two-sided F test with the null hypothesis that the slope is zero. (**g**) Bar plot of the number of soma-localized XRIs per neuron versus cytosolic GFP intensity for neurons in **e**. Bar height, median; error bar, interquartile range; gray dots, individual data points. n.s., not significant; ***, P < 0.0001; Kruskal-Wallis analysis of variance followed by post-hoc Dunn’s test. (**h-i**) Length (**h**) and width (**i**) of XRI versus time after AAV transduction (n = 14 XRIs from 8 neurons from 1 culture; length and width were normalized to the maximum values over time, respectively). Thick centerline, mean; shaded boundary, standard deviation; thin lines, data from individual XRIs. (**j**) A representative maximum intensity projection confocal image of CA1 neurons in the AAV-injected region of mice injected with AAV9-UBC-XRI-HA at CA1, allowed for expression for 14 days, and then fixed, sliced, and stained against NeuN for soma of neurons and against HA for XRI. Scale bar, 20 µm. (**k**) Histogram of the number of soma-localized XRIs per neuron in the field of view described in **j**, among NeuN-positive cells (n = 516 neurons from 1 field of view from 1 mouse). ‘96%’ with an arrow, 96% of the neurons had soma-localized XRI(s). (**l**) A representative confocal image of U2OS cells expressing XRI-HA for 4 days, taken after fixation, Nissl staining, and immunostaining against HA for XRI. Scale bar, 20 µm. See Supplementary Table 3 for details of statistical analysis.

We first set out to test whether human-engineered proteins known to self-assemble into filaments could be coaxed to reliably form continuously growing linear chains in cultured mammalian cells. We fused 14 human-designed filament-forming proteins (previously characterized in buffers, bacteria and yeast) to a short epitope tag (HA tag from hemagglutinin, for immunofluorescence imaging after protein expression and cell fixation) and expressed them in primary cultures of mouse hippocampal neurons (see Supplementary Table 1 for sequences of the motifs and Supplementary Table 2 for all tested constructs). Upon immunofluorescence staining, followed by imaging under confocal microscopy, two filament-forming proteins produced clear and stable fiber-like structures in the cytosol: 1POK(E239Y), a human-engineered filament-forming protein based on an *Escherichia coli* isoaspartyl dipeptidase^21^ (Fig. 1c,d) and DHF40, a computationally designed filament-forming protein^22^ (Extended Data Fig. 1a). The rest of the proteins produced unstructured aggregates, high nonassembly background and/or punctum-like structures in neurons (see Extended Data Fig. 1b for an example and Supplementary Table 2 for complete screening results). However, both filament-forming proteins also produced unstructured aggregates of protein in the cytosol. DHF40 showed a higher immunofluorescence background in cytosolic areas, which did not correspond either to fiber-like structures or unstructured aggregates, than did 1POK(E239Y), suggesting DHF40 had a higher level of free-floating protein monomers that did not bind to the protein assembly, than did 1POK(E239Y). Due to the lower immunofluorescence background, we selected 1POK(E239Y) as the filament-forming protein for further engineering in this study.

Because linear protein assembly would enable useful information encoding that could then be easily read out, we next performed protein engineering on 1POK(E239Y) to reduce the unstructured aggregates in cells. We reasoned that unstructured aggregates could be present due to unwanted lateral growth (Fig. 1e, left), as opposed to the longitudinal growth that would result in linear information encoding, and that reducing such lateral growth would discourage the formation of unstructured aggregates and thus encourage fiber-like linear protein assembly (Fig. 1e, right). We hypothesized that, by fusing a filament ‘insulator’ component to the lateral edge of the filament-forming monomer, unwanted lateral binding and growth of the protein assembly would be sterically blocked. We fused highly monomeric proteins that are used widely in bioengineering, monomeric enhanced green fluorescent protein (mEGFP)^23^ and maltose binding protein (MBP tag; an *E. coli* protein commonly used as a solubility tag for recombinant protein expression in mammalian^24^ and nonmammalian^25^ cells) to 1POK(E239Y) as insulators, together with the short epitope tag HA (Fig. 1c). We chose monomeric proteins as insulators because we reasoned that any homo-oligomeric binding of nonmonomeric proteins might encourage, rather than halt, unwanted lateral binding and growth of the protein assembly. Expression of these variants in mouse neurons showed that both produced only fiber-like structures, without any unstructured aggregates (Fig. 1d).

Next, we tested whether the mEGFP or MBP tag-bearing variants could encode information along their linear extent while preserving the temporal order of the information along their corresponding protein assemblies. If protein monomers with, for example, the epitope tag HA are constantly expressing, and the expression of protein monomers with, for example, the epitope tag FLAG are induced at a specific timepoint, then, at that timepoint, monomers with the FLAG tag will be more common than before, and thus added at a higher rate than before, along the growing protein chain. Then, the period of time at which FLAG is expressed could be read out easily via immunostaining against both HA and FLAG tags (Fig. 1f). We used the ERT2-iCre-ERT2-based chemically inducible Cre system^26^ to activate the expression of protein monomers with the FLAG tag, in a Cre-dependent FLEX vector, by 4-hydroxytamoxifen (4-OHT) treatment at defined times (Fig. 1f). Coexpressing these two vectors, both driven by the constitutive human ubiquitin (UBC) promoter, with a continuously expressed HA-bearing monomer in mouse neurons via DNA transfection, and then treating the neurons with 4-OHT for 15 min at a timepoint 2 days after transfection, was followed by fixing the neurons 1 day later, followed in turn by processing for immunofluorescence. We performed this experiment for each of the three variants: 1POK(E239Y), 1POK(E239Y)-mEGFP and 1POK(E239Y)-MBP (Fig. 1g). For the original 1POK(E239Y) variant without the insulator (Fig. 1g, left), we found a high similarity between the immunofluorescence patterns of the HA tag and the FLAG tag, showing that, as we had hypothesized, the 1POK(E239Y) variant could not preserve the temporal order of the protein monomers expressed (Fig. 1e). For the 1POK(E239Y)-mEGFP variant (Fig. 1g, middle), we also found a high similarity between the immunofluorescence patterns of the HA tag and the FLAG tag. We hypothesized that this might be due to the existence of a small, but non-negligible, unwanted lateral growth in this variant after 4-OHT treatment, so that newly expressed FLAG-fused monomers coated the lateral boundaries of the entire fiber assembly, resulting in uniform immunofluorescence of the FLAG tag along the assembly. For the 1POK(E239Y)-MBP variant, we found the immunofluorescence of the HA tag to show a continuous intensity profile along the protein assembly (Fig. 1g, right), while that of the FLAG tag showed higher intensity towards the two ends of the protein assembly and lower intensity towards the center of the protein assembly—a more polarized pattern. Thus, the 1POK(E239Y)-MBP variant showed a pattern that preserves temporal information created by the triggering of the FLAG tag at a defined point in time. We named this variant as the XRI, going forward throughout the rest of the study.

To characterize the electrophysiological integrity of neurons expressing XRIs, we made a bicistronic adeno-associated virus (AAV) construct containing mEGFP-P2A-XRI-HA, where P2A is a well-known self-cleaving peptide^27^ (Fig. 1h, upper left, schematic), so that cells expressing XRI could be identified by their green fluorescent protein (GFP) fluorescence for patch clamping. To our surprise, transduction of this bicistronic construct into cultured neurons resulted in not only mEGFP expression in the cytosol, but also a small amount of mEGFP decorating the XRI (Fig. 1h, lower left). The fluorescence intensity of mEGFP-decorated XRI in the GFP channel was dim—only about 60% higher than that of the non-XRI-occupied cytosol at the soma (Extended Data Fig. 1c,d). We reasoned that such slight XRI labeling by mEGFP was due to the self-cleaving efficiency of P2A being less than 100% (about 90% in mammalian cells)^27^, resulting in a small population of XRI monomers carrying mEGFP, and thus the presence of a sparse amount of mEGFP on the XRI assembly. Of GFP-positive neurons (that is, those with effective AAV-mediated gene delivery), about 80% had clear XRI formation at the soma 7 days after AAV transduction, with typically one to four XRIs at the soma (Extended Data Fig. 1e). Out of the remaining 20% of neurons that had no clear XRI formation at the soma, about half had punctum-like structures that did not have the clear, elongated shape of typical XRIs, and the other half had no resolvable structures (see the image of representative neurons in Extended Data Fig. 1c indicated by colored arrows). Using the cytosolic GFP intensity as an estimation of the expression level of the overall mEGFP-P2A-XRI bicistronic construct, we found that neurons with higher cytosolic GFP intensities had a significantly larger number of XRIs formed, and that neurons had unsuccessful XRI formation (of any shape) at very low cytosolic GFP intensities (Extended Data Fig. 1f,g). These results suggest that reliable XRI formation requires a sufficient expression level of XRI. We also injected the AAV of XRI-HA (without mEGFP) into the CA1 region of the mouse hippocampus and, after immunofluorescent staining of the HA tag and imaging, we found that 96% of CA1 neurons in the injected region had clear, successful XRI formation at the soma, 14 days post AAV injection, with typically one to four XRIs at the soma (Extended Data Fig. 1j,k).

We then used this bicistronic AAV construct to track XRI formation over time in live neurons, by imaging the GFP fluorescence in the same neurons daily for 7 days post-AAV transduction (Fig. 1h, right). We observed that XRI elongation during the 7 days was at a slightly increasing rate over time (Fig. 1i, normalized length of XRI versus time and Extended Data Fig. 1h, absolute length of XRI versus time). We also observed that the width of XRI increased during days 1–3 post AAV transduction, reaching a constant level from day 3 onwards (Fig. 1j, normalized width of XRI versus time and Extended Data Fig. 1i, absolute width of XRI versus time), raising the question of whether the blockage of lateral growth has a stochastic component that takes a few days to stabilize. Consistent with this initial stochasticity, we observed that no XRI structures appeared on day 1, about half of the XRIs appeared on day 2 and the remaining half appeared on day 3, post AAV transduction (Extended Data Fig. 1i), and that, before day 3, the XRIs were very short, at less than 10% of their lengths on day 7 post AAV transduction (Extended Data Fig. 1h). These observations suggested that the XRI system might only stabilize, and be able to record temporal information, starting around day 3 post AAV transduction (explored in experiments below).

**Extended Data Fig. 2:**
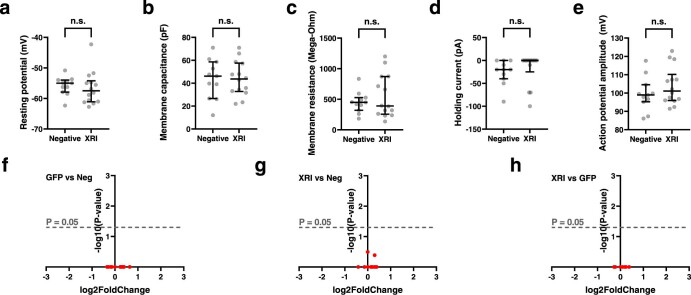
Electrophysiology and RNA-Seq analysis of cultured neurons expressing XRI. (**a-e**) Scatter plots of the electrophysiological properties of cultured neurons with and without XRI expression, in terms of (**a**) resting potential, (**b**) membrane capacitance, (**c**) membrane resistance, (**d**) holding current while held at −65 mV, and (**e**) action potential amplitude. Middle line, median; error bar, interquartile range; gray dots, individual data points. Cultured mouse hippocampal neurons (the ‘XRI’ group) were transduced with AAV9-UBC-mEGFP-P2A-XRI-HA as described in Fig. 1h on day in vitro 7 (DIV 7) for electrophysiological characterization via whole-cell patch clamp on DIV 14-16 side-by-side with neurons without AAV transduction (the ‘Negative’ group). Neurons with XRIs were identified by fluorescence imaging in the GFP channel before whole-cell patch clamp. n = 11 neurons from 3 cultures for the Negative group; n = 14 neurons from 4 cultures for the XRI group; n.s., not significant; two-sided Wilcoxon rank sum test. See Supplementary Table 3 for details of statistical analysis. (**f-h**) Differential expression analysis across mouse genes via RNA-Seq, comparing neuron cultures expressing GFP via AAV transduction (the ‘GFP’ group; n = 14 neuron cultures) with neuron cultures without AAV transduction (the ‘Neg’ group; n = 17 neuron cultures) as the baseline group (**f**), neuron cultures expressing XRI via AAV transduction (the ‘XRI’ group; n = 24 neuron cultures) with the ‘Neg’ group as the baseline group (**g**), or the ‘XRI’ group with the ‘GFP’ group as the baseline group (**h**). Results plotted as scatter plots (one dot for one gene) in **f-h** of the fold change over the baseline group of the transcript count on a logarithmic scale to base 2 (log2FoldChange) versus the P-value for the null hypothesis is that there is no differential expression across the two groups plotted in the format of -log10(P-value), using two-sided Wald test with Benjamini-Hochberg correction. See Supplementary Table 4 for full results from the differential expression analysis. Mouse hippocampal neuron cultures were randomly split into the ‘Neg’ group, the ‘GFP’ group transduced with AAV9-UBC-GFP on day in vitro 7 (DIV 7), and the ‘XRI’ group transduced with AAV9-UBC-XRI-HA on DIV 7. On DIV 14, RNA was extracted from individual neuron cultures and then cDNA was generated from RNA and sequenced on an Illumina MiSeq. Sequences were mapped to the GRCm38 (mm10) reference genome (with gene annotations obtained from Ensembl) and gene expression raw counts were normalized and batch-effect adjusted using DESeq2^53^, followed by differential expression analysis and statistics using DESeq2.

**Extended Data Fig. 3:**
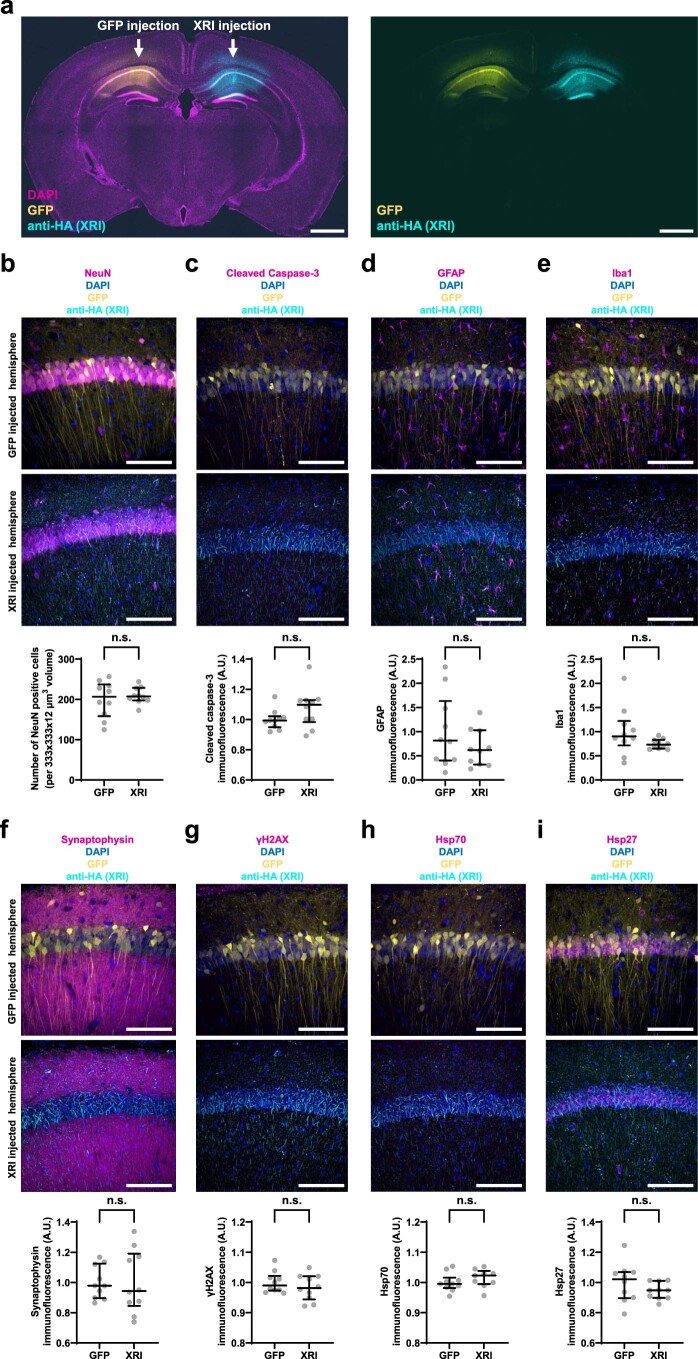
Immunohistochemical characterization of cellular and synaptic state markers in mouse brains expressing XRI. (**a**) Representative confocal images of brain slices from adult mice expressing GFP (or XRI) at CA1 region in the left (or right) cerebral hemisphere following AAV injection. 3-month-old mice were injected with AAV9-GFP at the CA1 region in the hippocampus of the left cerebral hemisphere and injected with AAV9-XRI-HA at the CA1 region in the hippocampus of the right cerebral hemisphere. When the mice reached 14 days after AAV injection, they were euthanized, perfused with 4% PFA, and brains were sliced coronally at 50 μm in 1X PBS, and stained with antibodies against one of the cellular and synaptic markers below (see **b-i**) and against HA tag to label XRIs, together with DAPI to label nuclei. Staining intensities of cellular and synaptic markers in the cortex (or CA1) were imaged volumetrically using a 40x objective on a spinning disk confocal microscope, with identical imaging conditions, measured in ImageJ as the averaged fluorescent intensities of the fluorescent secondary antibodies over imaged fields of view (333 μm × 333 μm × 50 μm for each fields of view), and compared between the left hemisphere and the right hemisphere. Scale bars, 1 mm. (**b-i**, top) Representative confocal images of cortex and CA1 in the GFP-injected hemisphere and XRI-injected hemisphere in the brain slices stained with antibodies against each of the cellular and synaptic markers indicated, and DAPI. Scale bars, 100 μm. (**b-i**, bottom) Scatter plots of the staining intensities for each of the cellular and synaptic markers between the GFP-injected hemisphere and XRI-injected hemisphere; for each marker in each hemisphere, n = 10 fields of view (FOVs) from 5 mice (2 FOVs for the GFP-injected hemisphere and 2 FOVs for the XRI-injected hemisphere per mouse). Middle line, median; error bar, interquartile range; gray dots, individual data points. n.s., not significant; two-sided Wilcoxon rank sum test. See Supplementary Table 3 for details of statistical analysis. (**b**) NeuN (a neuronal marker). (**c**) Cleaved Caspase-3 (an apoptotic marker). (**d**) GFAP (an astrocyte marker). (**e**) Iba1 (a microglial marker). (**f**) Synaptophysin (a synaptic protein marker). (**g**) γH2AX (a DNA damage marker). (**h,i**) Hsp70 and Hsp27 (cell physiological stress markers).

Next, we performed electrophysiology and RNA-sequencing (RNA-seq) analysis of cultured neurons expressing XRI, and observed that XRI expression does not alter the electrophysiology and endogenous gene expression in these neurons (Extended Data Fig. 2 and see Supplementary Table 4 for full results from the RNA-seq differential expression analysis across mouse genes). We further performed immunohistochemical characterization of mouse brains expressing XRI and found XRI expression in cell populations in vivo does not alter cellular and synaptic state markers, including NeuN as a neuronal marker, cleaved Caspase-3 as an apoptotic marker, GFAP as an astrocyte marker, Iba1 as a microglial marker, Synaptophysin as a synaptic protein marker, γH2AX as a DNA damage marker and Hsp70 and Hsp27 as cell physiological stress markers (Extended Data Fig. 3). Since our primary focus was to develop and apply recording systems in postmitotic cells such as neurons, we did not focus on XRI usage in dividing cells, but did note that expression of the current XRI in dividing cells encountered difficulty (Extended Data Fig. 1l), with XRI-like structures forming, but accompanied by aggregate-like structures. Thus, we retained our focus on nondividing cells, specifically, neurons.

**Fig. 2:**
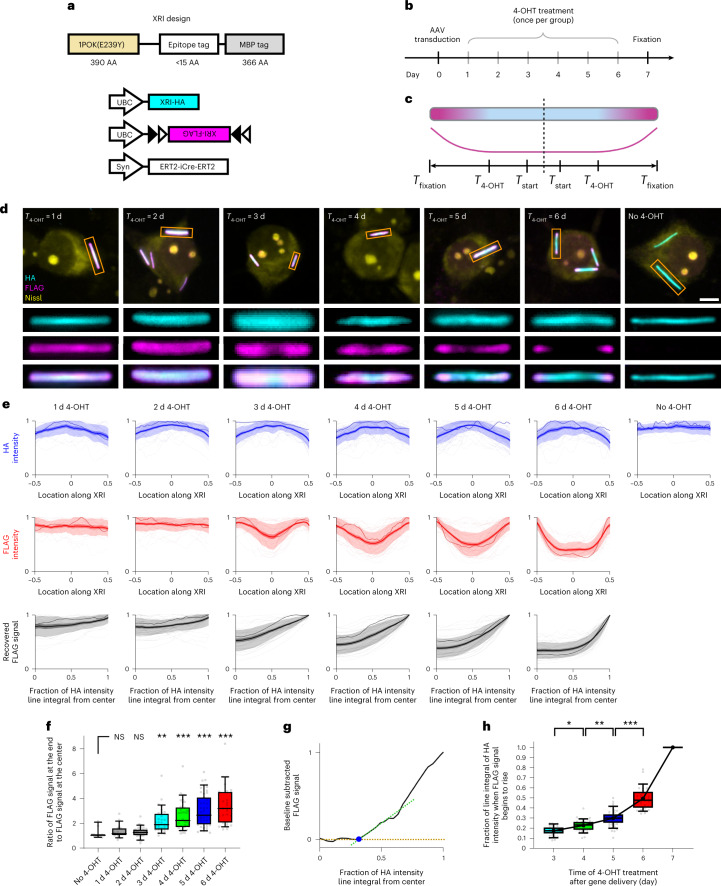
Characterization and calibration of XRIs via timed chemically induced expression. **a**–**c**, Schematics of the constructs cotransduced into neurons (**a**), experiment pipeline (**b**) and expected epitope distribution along the XRI protein self-assembly (**c**) in the chemically induced gene expression experiment. XRI-HA, XRI with the epitope tag HA; XRI-FLAG, XRI with the epitope tag FLAG. The constructs were delivered to cells on day 0 via AAV transduction, and fixed 7 days later (*T*_fixation_ = 7 days). *T*_4-OHT_, time of 4-OHT treatment (once only per group of neurons); *T*_*s*tart_, the time at which XRI starts recording information after gene delivery and expression of XRI (see **f** below, where *T*_start_ is measured to be 3 days after AAV transduction). **d**, Representative confocal images of cultured mouse hippocampal neurons expressing constructs in **a**, taken after fixation, Nissl staining and immunostaining against HA and FLAG tags. Three rows of rectangular panels at the bottom, enlarged views of regions marked in orange rectangles in the top row of square panels. Scale bar, 5 µm. **e**, HA intensity profile along the XRI (top row), FLAG intensity profile along the XRI (middle row) and recovered FLAG signal (by averaging the two FLAG intensity profiles from the two halves of the XRIs) plotted against the fraction of the line integral of HA intensity (a value between 0 and 1; 0 corresponds to the center of the XRI, and 1 corresponds to the end of the XRI; bottom row), from the experiment described in **a**–**c** (*n* = 21 XRIs from 13 neurons from two cultures for ‘1d 4-OHT’ group; *n* = 37 XRIs from 19 neurons from two cultures for ‘2d 4-OHT’ group; *n* = 32 XRIs from 22 neurons from two cultures for ‘3d 4-OHT’ group; *n* = 38 XRIs from 22 neurons from two cultures for ‘4d 4-OHT’ group; *n* = 47 XRIs from 32 neurons from two cultures for ‘5d 4-OHT’ group; *n* = 29 XRIs from 19 neurons from two cultures for ‘6d 4-OHT’ group; *n* = 11 XRIs from 5 neurons from two cultures for ‘No 4-OHT’ group). Each raw trace was normalized to its peak to show relative changes before averaging; see Extended Data Fig. 4a for HA intensity profile, FLAG intensity profile and recovered FLAG signal before normalization. Thick centerline, mean; darker boundary in the close vicinity of the thick centerline, s.e.m.; lighter boundary, s.d.; lighter thin lines, data from individual XRIs; darker thin line, data from the corresponding XRI in the orange rectangle in **d**. See Extended Data Fig. 5 for the detailed process flow of extracting signals from XRI assemblies. **f**, Box plot of the ratio of the FLAG signal at the end of XRI to the FLAG signal at the center of XRI. Middle line in box plot, median; box boundary, interquartile range; whiskers, 10–90 percentile; minimum and maximum, not indicated in the box plot; gray dots, individual datapoints. NS, not significant; **P = 0.0028; ***P < 0.0001; Kruskal–Wallis analysis of variance followed by post hoc Dunn’s test with ‘No 4-OHT’ as control group. **g**, An example line plot of the FLAG signal plotted against the fraction of line integral of HA intensity (from the ‘5d 4-OHT’ group in **e**), showing the quantification of the fraction of line integral of HA intensity when FLAG signal begins to rise (blue dot). Gold dashed line, the FLAG signal at the center of XRI (as baseline); green dashed line, a line fitted to the initial rising phase of the FLAG signal (defined as the portion of FLAG signal between 10% to 50% of the peak FLAG signal); blue dot, intersection of the two dashed lines. **h**, Fraction of line integral of HA intensity when FLAG signal begins to rise plotted against the time of 4-OHT treatment after gene delivery, for XRIs in **g**. The line integral of HA intensity was normalized to ‘1’ for day 7, the time of cell fixation and thus the end of XRI growth. Middle line in box plot, median; box boundary, interquartile range; whiskers, 10–90 percentile; minimum and maximum, not indicated in the box plot; small gray dots, individual datapoints; large black dot, mean; black line, linear interpolation of the means. *P = 0.0378; **P = 0.0032; ***P < 0.0001; Kruskal–Wallis analysis of variance followed by post hoc Dunn’s test. See Supplementary Table 3 for details of statistical analysis.

**Extended Data Fig. 4:**
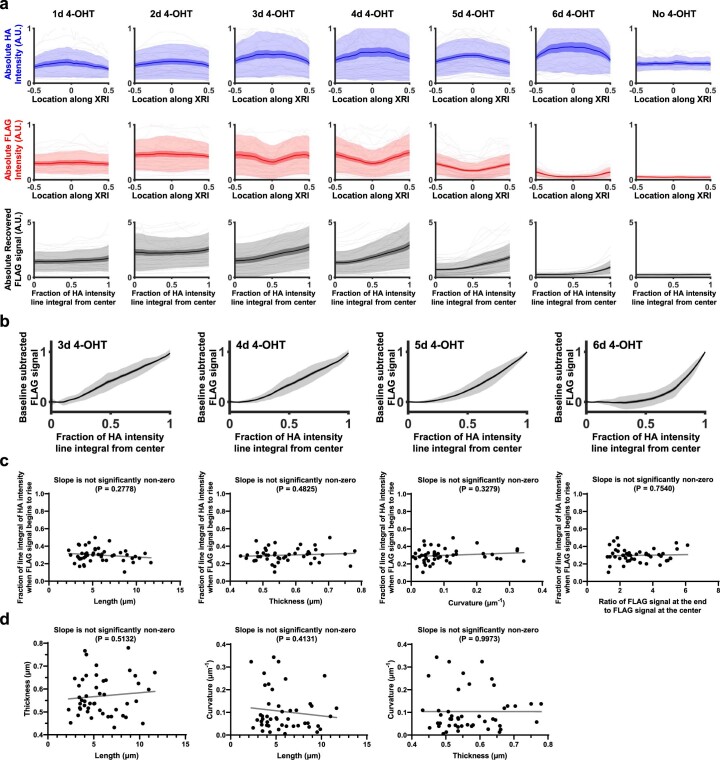
Additional intensity profile analysis and geometric analysis of XRI. (**a**) Absolute HA intensity profile along the XRI (top row), absolute FLAG intensity profile along the XRI (middle row), and absolute recovered FLAG signal (by averaging the two FLAG intensity profiles from the two halves of the XRIs) plotted against the fraction of the line integral of HA intensity (a value between 0 and 1; 0 corresponds to the center of the XRI, and 1 corresponds to the end of the XRI; bottom row), from the experiment described in Fig. 2a-c (n = 21 XRIs from 13 neurons from 2 cultures for ‘1d 4-OHT’ group; n = 37 XRIs from 19 neurons from 2 cultures for ‘2d 4-OHT’ group; n = 32 XRIs from 22 neurons from 2 cultures for ‘3d 4-OHT’ group; n = 38 XRIs from 22 neurons from 2 cultures for ‘4d 4-OHT’ group; n = 47 XRIs from 32 neurons from 2 cultures for ‘5d 4-OHT’ group; n = 29 XRIs from 19 neurons from 2 cultures for ‘6d 4-OHT’ group; n = 11 XRIs from 5 neurons from 2 cultures for ‘No 4-OHT’ group). Thick centerline, mean; darker boundary in the close vicinity of the thick centerline, standard error of mean; lighter boundary, standard deviation; lighter thin lines, data from individual XRIs. (**b**) Baseline subtracted FLAG signal plotted against the fraction of the line integral of HA intensity for the ‘3d 4-OHT’, ‘4d 4-OHT’, ‘5d 4-OHT’, ‘6d 4-OHT’ groups in Fig. 2e. Thick centerline, mean; darker boundary in the close vicinity of the thick centerline, standard error of mean; lighter boundary, standard deviation. (**c**) Scatter plots of the fraction of the line integral of HA intensity when the FLAG signal begins to rise versus the length of the XRI, the thickness of the XRI, the curvature of the XRI, and the ratio of the FLAG signal at the end to the FLAG signal at the center, for XRIs in neurons in the ‘5d 4-OHT’ group in Fig. 2 (the ‘5d 4-OHT’ group was randomly chosen for this analysis; n = 47 XRIs from 32 neurons from 2 cultures). Gray line, line fit from linear regression; P value, two-sided F test with the null hypothesis that the slope is zero. (**d**) Scatter plot of the thickness of XRI versus the length of XRI, the curvature of XRI versus the length of XRI, and the curvature of XRI versus the thickness of XRI, for XRIs in **c**. Gray line, line fit from linear regression; P value, two-sided F test with the null hypothesis that the slope is zero.

To study how accurate this XRI protein assembly could preserve time information, we again used the chemically inducible Cre system and treated different neuron cultures expressing XRI with 4-OHT at different times after beginning of expression. We used AAVs to deliver the chemically inducible Cre system and the XRI genes into cultured mouse neurons and allowed a 7-day expression time window before fixation, immunofluorescent labeling and imaging. We divided the neuron cultures into seven groups and performed 4-OHT treatment at 1, 2, 3, 4, 5 or 6 days after AAV transduction, or not at all (Fig. 2a–c). We found continuous HA immunofluorescence in neurons in all groups (Fig. 2d). We found XRI assemblies to have no FLAG immunofluorescence in neurons without 4-OHT treatment, indicating negligible leak expression of the chemically inducible Cre system (Fig. 2d, ‘No 4-OHT’ panel). We found the FLAG immunofluorescence to have strong polarized patterns (for example, brighter at the ends than in the middle) in neurons with 4-OHT treatment on days 3, 4, 5 or 6 after AAV transduction, but not to have strongly polarized patterns in neurons with 4-OHT treatment on day 1 or 2 after AAV transduction (Fig. 2d,e and see Extended Data Fig. 4a for the unnormalized version of the plots in Fig. 2e); the HA tag showed a gentle polarization trend in the opposite direction, perhaps because the HA-bearing subunits available were landing on the growing protein chain at greater distances than before, due to the FLAG-bearing subunits having already been added.

**Extended Data Fig. 5:**
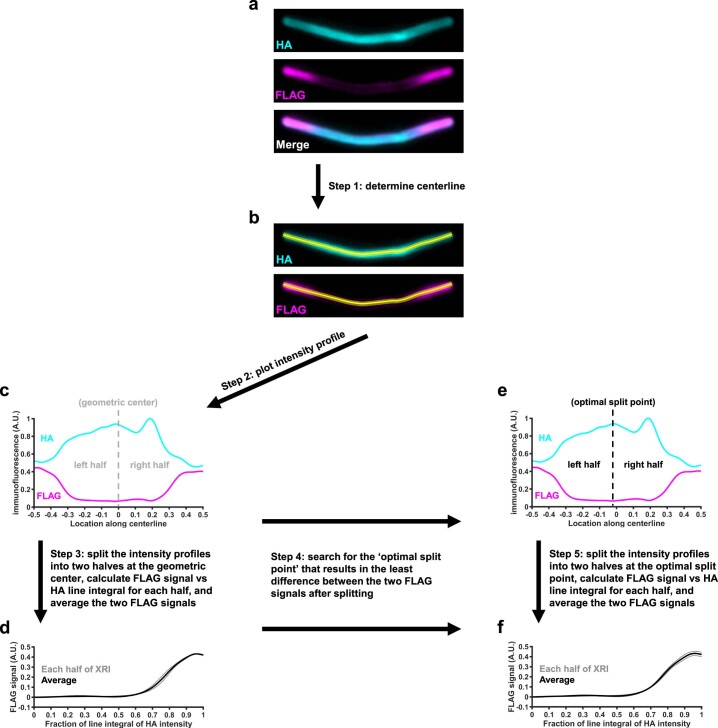
Process flow for extracting information from XRI assemblies. **Step 1:** For each XRI (**a**), a curved centerline was drawn along the longitudinal axis of the XRI in the anti-HA channel (**b**). The centerline width was set to half of the width of the XRI. **Step 2:** The intensity profiles along this centerline were measured in the anti-HA channel (resulting in an HA line profile; cyan curve in **c**) and in the other XRI epitope staining channel, such as in the anti-FLAG channel (resulting in a FLAG line profile; magenta curve in **c**). **Step 3:** Next, each of the line profiles was split into two half line profiles using the geometric center point of the XRI (the 50% length point along the centerline, measuring from the end of the XRI; gray dashed vertical line in **c**) as the ‘split point’. Each of the half HA line profiles was then converted into a line integral of HA, by integrating the line profile with respect to the distance along the half centerline starting from the split point, and then these line integrals of HA were normalized to the maximum integral value so that each line integral of HA started at the value 0 at the split point of the XRI, and gradually increased to the value 1 at the end of the XRI (see **Methods** for equations for the quantifications throughout this figure). For the corresponding half FLAG line profiles, line integrals were also calculated but not normalized. At this point, we have the line integrals of HA and FLAG, which correspond to the cumulative HA and FLAG intensities along each half of the XRI. The FLAG intensity change per unit change in the cumulative HA intensity, defined as the FLAG signal, was calculated by taking the derivative of the line integral of FLAG with respect to the line integral of HA (gray curves in **d**). At this stage, we had obtained the line integral of HA and the FLAG signal from each of the halves of the XRI, and the final extracted FLAG signal from this XRI (black curves in **d**) was defined as the point-by-point average of the two FLAG signals from the two halves of the XRI. **Step 4:** We found the two obtained FLAG signals from the same XRI have small but noticeable differences (see the two gray curves in **d**). We reasoned that such small but noticeable discrepancies between the two halves of the same XRI was due to the asymmetry of the XRI, and the choice of the exact geometric center as the split point may not be optimal. To minimize the discrepancy between the two FLAG signals from the two halves of the same XRI, we searched for an optimal split point (black dashed vertical line in **e**) near the geometric center of the XRI (searching range was the geometric center ±25% of the total XRI length, that is, between −0.25 and 0.25 on the x-axis in **e**), so that using this optimal split point, instead of the geometric center, as the split point would result in the least difference (in terms of sum of squared differences) between the two FLAG signals from the two halves of the split XRI. **Step 5:** Same as **Step 3**, except that the optimal split point, instead of the geometric center, was used to split the line profiles into two halves (**f**). We found the resulting final FLAG signal (after averaging those from the two halves) when using the geometric center as the split point was similar to that when using the optimal split point as the split point (compare the black line in **d** and **f**). Nevertheless, we used the optimal split point as the split point to analyze XRIs throughout this paper.

Next, we quantified the relationship between the times of 4-OHT treatment and the resulting FLAG immunofluorescence patterns on XRI assemblies in neurons. Because the XRI growth is bidirectional over the 7-day experiment, we defined the fractional cumulative HA expression (that is, the normalized, unidirectional line integral of HA immunofluorescence starting outwards from the center of the XRI) at the center of the XRI as ‘0’ and at the end of the XRI as ‘1’ (see Extended Data Fig. 5 for details of quantification). We hypothesized that this measure, the fractional cumulative HA expression, would correspond to a calibratable measure of time, postulating HA-bearing monomers to be added to the protein chain at a rate independent of the presence of non-HA-bearing monomers (that is, FLAG-bearing monomers here), at least over the timescale of this experiment. That is, when FLAG-bearing monomers are being created, HA-bearing monomers are still being added to the growing protein chain at their own rate, although they are landing at more distant places along the chain, because FLAG-bearing monomers that were already added to the chain would lengthen the distance at which new HA-bearing monomers would land. Is this a reasonable postulate? We did see HA intensity to decrease considerably towards the end of XRI, when FLAG intensity increased due to 4-OHT induced expression of FLAG-bearing monomers (‘3-6d 4-OHT’ groups the first row in Fig. 2e). In addition, this decrease in HA intensity towards the end of XRI was not observed without 4-OHT treatment (‘No 4-OHT’ group in the first row in Fig. 2e). Because the 1POK(E239Y)-mediated fiber assembly has a fixed longitudinal monomer-to-monomer distance (around 4 nm from electron microscopy measurements)^21^, the above results suggest that FLAG-bearing monomers took over a considerable amount of longitudinal space at the end of XRI and thus diluted the line density of HA-bearing monomers.

This raises the question: is the assumption that HA-bearing and FLAG-bearing monomers are adding independently, each at a rate independent of the presence of the other monomer, a good one? If the binding and retention of HA-bearing monomers and FLAG-bearing monomers onto the XRI are both rare enough in time, that the chance of both types of monomers competing for the same slot on the XRI is negligible, then this would be plausible. And, in this case, the fractional cumulative HA expression would still be a proper, calibratable measure of time. That is, if units with a new tag are supplementing the units being constitutively synthesized bearing an old tag, the latter units would not be added at a slower rate (that is, there is no competition between the new units and the old units for being added to the growing chain), but instead would be added at the same rate, simply being spaced out further from each other, separated by the units bearing the new tag. This would make the line integral the appropriate measure for extracting absolute time measurements. We sought to empirically test the hypothesis that absolute time measurements could be extracted from this specific measure. We averaged the FLAG signals across the two halves of the XRI (since XRIs are symmetric), to obtain the final FLAG signal (Fig. 2e, bottom). Then, we calculated the ratio of the FLAG signal at the end of the XRI to the FLAG signal at the center of the XRI (Fig. 2f), confirming that the polarized patterns of FLAG immunofluorescence on XRIs are present in neurons with 4-OHT treatments 3, 4, 5 or 6 days after AAV transduction, but not in neurons with 4-OHT treatments 1 or 2 days after AAV transduction, as hypothesized above in the section on time-lapse imaging. Therefore, we further analyzed the XRIs in neurons with 4-OHT treatments 3, 4, 5 or 6 days after AAV transduction, to characterize the relationship between the time of 4-OHT treatment and the fraction of the line integral of HA intensity at which the FLAG signal began to rise.

**Extended Data Fig. 6:**
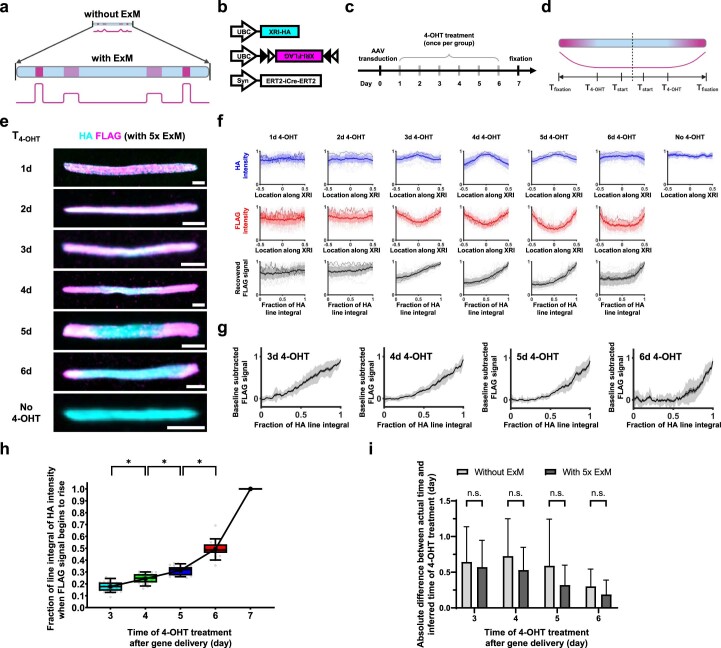
Imaging XRIs using expansion microscopy. We replicated the experiment in Fig. 2 and applied expansion microscopy^28^ (ExM) instead of confocal microscopy for immunofluorescence imaging of XRIs. We optimized the digestion methods (removing the proteinase K digestion step and replacing it with a heat-based softening step) starting from the TREx^54^ ExM protocol, while receiving inspirations from the ExR^55^ protocol, to achieve uniform expansion of XRI assemblies and post-expansion immunostaining/immunofluorescence at a high signal-to-noise-ratio (at a linear expansion factor of ~ 5x), with antibody staining against NeuN to locate the somata of neurons. (**a**) Schematic of using expansion microscopy (ExM) to increase the spatial resolution of immunofluorescence imaging. (**b**-**d**) Schematics of the constructs co-transduced to neurons (**b**), experiment pipeline (**c**), and expected epitope distribution along the XRI protein self-assembly (**d**) in the chemically induced gene expression experiment, as in Fig. 2. (**e**) Representative confocal images of XRIs in cultured mouse hippocampal neurons expressing constructs in **b** with different times of 4-OHT treatment (T_4-OHT_), after 5x ExM. Scale bars, 5 µm after ExM (equivalent to 1 µm in biological units, for example when divided by the expansion factor). (**f**) HA intensity profile along the XRI (top row), FLAG intensity profile along the XRI (middle row), and recovered FLAG signal (by averaging the two FLAG intensity profiles across the two halves of XRI), plotted against the fraction of the line integral of HA intensity (a value between 0 and 1; 0 corresponds to the center of XRI, and 1 corresponds to the end of XRI; bottom row), from the experiment described in **a**-**d** (n = 32 XRIs from 19 neurons from 2 cultures for ‘1d 4-OHT’ group; n = 30 XRIs from 16 neurons from 2 cultures for ‘2d 4-OHT’ group; n = 23 XRIs from 14 neurons from 2 cultures for ‘3d 4-OHT’ group; n = 24 XRIs from 15 neurons from 3 cultures for ‘4d 4-OHT’ group; n = 22 XRIs from 17 neurons from 3 cultures for ‘5d 4-OHT’ group; n = 19 XRIs from 15 neurons from 3 cultures for ‘6d 4-OHT’ group; n = 7 XRIs from 3 neurons from 1 culture for ‘No 4-OHT’ group). Each raw trace was normalized to its peak to show relative changes before averaging. Thick centerline, mean; darker boundary in the close vicinity of the thick centerline, standard error of mean; lighter boundary, standard deviation; lighter thin lines, data from individual XRIs; darker thin line, data from the corresponding XRI in **e**. See Extended Data Figure 5 for the detailed process flow of extracting signals from XRI assemblies. (**g**) Baseline subtracted FLAG signal plotted against the fraction of the line integral of HA intensity for the ‘3d 4-OHT’, ‘4d 4-OHT’, ‘5d 4-OHT’, ‘6d 4-OHT’ groups in **f**. Thick centerline, mean; darker boundary in the close vicinity of the thick centerline, standard error of mean; lighter boundary, standard deviation. (**h**) Fraction of line integral of HA intensity when FLAG signal begins to rise, plotted against the time of 4-OHT treatment after gene delivery, for XRIs in **g**. The line integral of HA intensity was normalized to ‘1’ for day 7, the time of cell fixation and thus the end of XRI growth. Middle line in box plot, median; box boundary, interquartile range; whiskers, 10-90 percentile; minimum and maximum, not indicated in the box plot; small gray dots, individual data points; large black dot, mean; black line, linear interpolation of the means. *, P < 0.05 (from left to right, P = 0.0269, 0.0164, and 0.0123); Kruskal-Wallis analysis of variance followed by post-hoc Dunn’s test. See Supplementary Table 3 for details of statistical analysis. n = 23 XRIs from 14 neurons from 2 cultures for ‘3d 4-OHT’ group; n = 24 XRIs from 15 neurons from 3 cultures for ‘4d 4-OHT’ group; n = 22 XRIs from 17 neurons from 3 cultures for ‘5d 4-OHT’ group; n = 19 XRIs from 15 neurons from 3 cultures for ‘6d 4-OHT’ group. (**i**) Bar plot of the absolute difference between the actual time and the inferred time of 4-OHT treatment, without and with 5x ExM. For each XRI, the inferred time of 4-OHT treatment was calculated from the fraction of the line integral of HA intensity when the FLAG signal begins to rise, using the black line in Fig. 2h (for XRI without ExM) or the black line in **h** (for XRI with 5x ExM) as time calibration. Bar height, mean; error bar, standard deviation. n.s., not significant; Bonferroni corrected two-sided Wilcoxon rank sum tests. For XRI without ExM, n = 32 XRIs from 22 neurons from 2 cultures for ‘3d 4-OHT’ group; n = 38 XRIs from 22 neurons from 2 cultures for ‘4d 4-OHT’ group; n = 47 XRIs from 32 neurons from 2 cultures for ‘5d 4-OHT’ group; n = 29 XRIs from 19 neurons from 2 cultures for ‘6d 4-OHT’ group; for XRI with 5x ExM, n = 23 XRIs from 14 neurons from 2 cultures for ‘3d 4-OHT’ group; n = 24 XRIs from 15 neurons from 3 cultures for ‘4d 4-OHT’ group; n = 22 XRIs from 17 neurons from 3 cultures for ‘5d 4-OHT’ group; n = 19 XRIs from 15 neurons from 3 cultures for ‘6d 4-OHT’ group.

To quantify the fraction of the line integral of HA intensity at which the FLAG signal began to rise, we generated the net waveform of the FLAG signal with respect to the fraction of the line integral of HA intensity, by subtracting the baseline (that is, the FLAG signal when the fraction of the line integral of HA intensity is zero) from the FLAG signal (Extended Data Fig. 4b). Next, we extrapolated the initial rising phase of the FLAG signal (defined as the period over which the FLAG signal increased from 10% to 50% of its peak value) until it intersected the prerising phase baseline (Fig. 2g). The fraction of the HA line integral at this intersection point was defined as the point in time (although of course, to pinpoint a numerical value for the time requires calibration, discussed below) at which the FLAG signal began to rise. Importantly, this point did not depend on the length, thickness or curvature of the XRI, nor did it change with the precise value of the ratio of the FLAG signal at the end of the XRI to the FLAG signal at the center of the XRI (Extended Data Fig. 4c), implying that this measure of time was a robust measure, and not dependent on the details of the geometry of the XRI or any associated constraints on the formation of the XRI. We also did not observe any correlation between the length, thickness and curvature of XRI (Extended Data Fig. 4d), implying a certain degree of robustness as to the independence of different XRI geometrical attributes, and consistent with the stabilization hypothesis above. As the time of 4-OHT treatment time increased, the fraction of the line integral of HA intensity when the FLAG signal began to rise also increased, albeit at a nonconstant (that is, increasing) rate, suggesting that the expression rate of AAV delivered XRI genes, and the elongation rate of XRI, increased over time (Fig. 2h). These results are in agreement with the earlier observation in the time-lapse experiment, above, where the elongation of XRI growth pattern (compare Fig. 1i with Fig. 2h). These observations are consistent with the idea that the rate of addition of HA-bearing monomers to the XRI assembly was not altered by the presence of the FLAG-bearing monomers over the timescale measured in our experiments, although we do not know whether such independence was indeed due to the two kinds of monomers rarely competing in time for the same slot on the XRI (as we speculated in the previous paragraph) or due to other mechanisms. Nevertheless, we found the time of a given cellular event can indeed be extracted from XRI geometry and label density, analyzed thus. We normalized this value to be 1 on day 7, because that was the time of cell fixation and thus the end of XRI growth (see day 7 in Fig. 2h). We also replicated this experiment and applied expansion microscopy^28^ (ExM) instead of confocal microscopy for immunofluorescence imaging of XRI, obtaining similar results (Extended Data Fig. 6). Thus, the predictable relationship between time of drug administration, and the fraction of the line integral of the HA intensity at which the FLAG signal began to increase, enables us to calibrate time information in XRI data analysis.

**Fig. 3:**
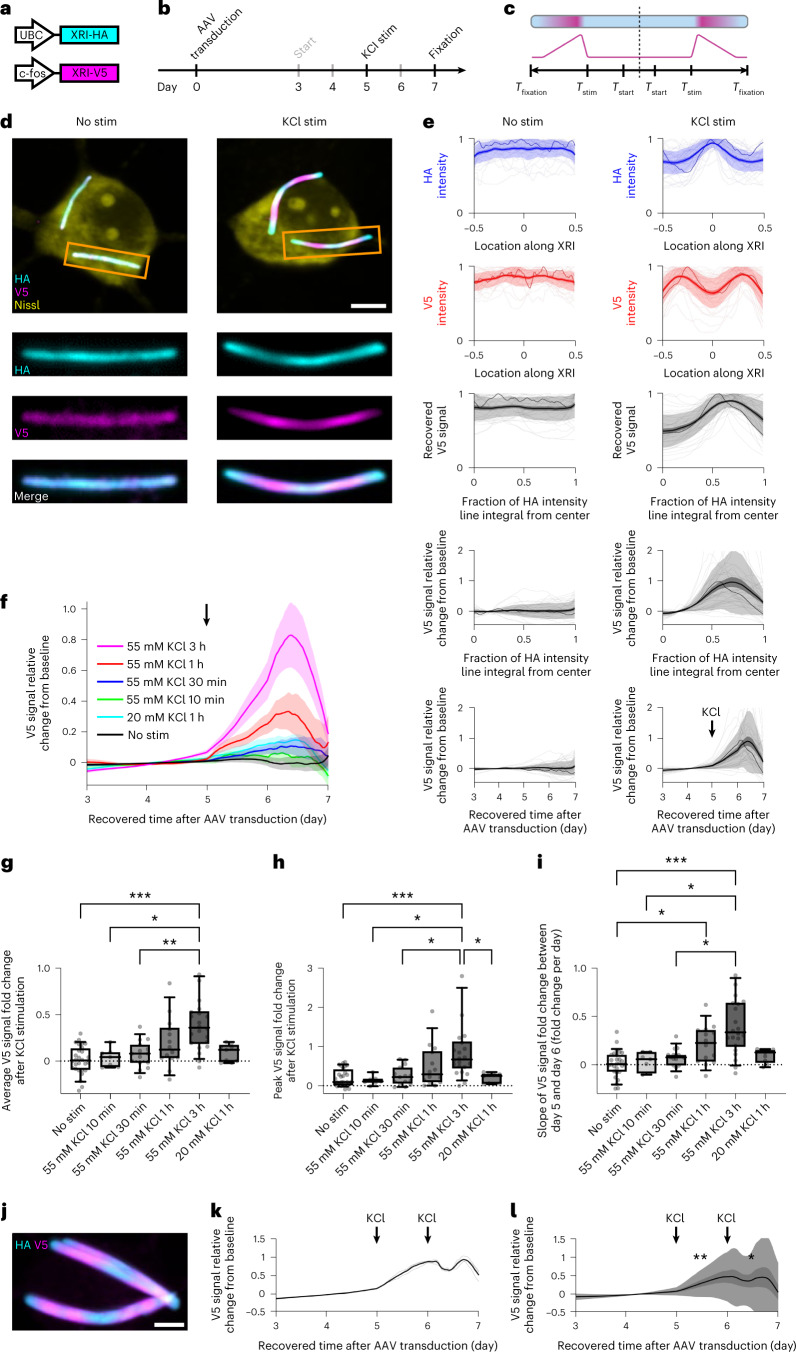
Recording the timecourse of c-fos-promoter-driven expression with XRI. **a**–**c**, Schematics of the AAV constructs cotransduced to neurons (**a**), experiment pipeline (**b**) and expected epitope distribution along the XRI protein self-assembly (**c**) in the c-fos-promoter-driven gene expression experiment. XRI-HA, XRI with the epitope tag HA; XRI-V5, XRI with the epitope tag V5; c-fos, c-fos promoter; *T*_stim_, the time of the onset of stimulation of neuron activity by KCl; *T*_start_, the time at which XRI starts recording information after gene delivery and expression of XRI, which is measured to be 3 days after AAV transduction in Fig. 2f. **d**, Representative confocal images of cultured mouse hippocampal neurons expressing constructs in **a**, taken after fixation, Nissl staining and immunostaining against HA and V5 tags. KCl stim, 55 mM KCl stimulation for 3 h starting at *T*_stim_ = 5 days; three rows of rectangular panels at the bottom, enlarged views of regions marked in orange rectangles in the top row of square panels. Scale bar, 5 µm. **e**, HA intensity profile along the XRI (first row), V5 intensity profile along the XRI (second row), recovered V5 signal (calculated from the intensity profiles) plotted against the fraction of the line integral of HA intensity (third row), V5 signal relative change from baseline (ratio of the V5 signal to the V5 signal at the center of the XRI, and then minus 1) plotted against the fraction of the line integral of HA intensity (fourth row) and V5 signal relative change from baseline plotted against recovered time after AAV transduction (using the black line in Fig. 2h as time calibration for time recovery from the line integral of HA intensity; fifth row), from the experiment described in **a**–**c** (*n* = 30 XRIs from 28 neurons from two cultures for ‘No Stim’ group; *n* = 40 XRIs from 22 neurons from three cultures for ‘KCl Stim’ group). Thick centerline, mean; darker boundary in the close vicinity of the thick centerline, s.e.m.; lighter boundary, s.d.; lighter thin lines, data from individual XRIs; darker thin line, data from the corresponding XRI in the orange rectangle in **d**. In the first three rows, each raw trace was normalized to its peak to show relative changes before averaging. See Extended Data Fig. 5 for the detailed process flow of extracting signals from XRI assemblies. **f**, V5 signal relative change from baseline plotted against recovered time after AAV transduction from XRIs in neurons under different KCl stimulations at *T*_stim_ = 5 days (black arrow, onset of KCl stimulation; *n* = 22 neurons from three cultures for ‘55 mM KCl 3 h’ group; *n* = 14 neurons from four cultures for ‘55 mM KCl 1 h’ group; *n* = 15 neurons from two cultures for ‘55 mM KCl 30 min’ group; *n* = 7 neurons from one culture for ‘55 mM KCl 10 min’ group; *n* = 9 neurons from one culture for ‘20 mM KCl 1 h’ group; *n* = 28 neurons from two cultures for ‘No Stim’ group;). Centerline, mean; shaded boundary, standard error of mean. **g**–**i**, Box plot of the average V5 signal relative change from baseline over time between day 5 and day 7 (that is, within 48 h after the onset time of KCl stimulation) (**g**), the peak V5 signal relative change from baseline over time between day 5 and day 7 (**h**) and the slope of V5 signal relative change over time from baseline between day 5 and day 6 (**i**) for neurons in **f**. Middle line in box plot, median; box boundary, interquartile range; whiskers, 10–90 percentile; minimum and maximum, not indicated in the box plot; gray dots, individual datapoints. *P < 0.05; **P < 0.01; ***P < 0.001; Kruskal–Wallis analysis of variance followed by post hoc Dunnʼs tests between every two groups; test result was not significant for a pair without *, ** or *** indicated. **j**, A representative confocal image of XRIs in a cultured mouse hippocampal neuron expressing constructs in **a**, taken after fixation (7 days after AAV transduction), Nissl staining and immunostaining against HA and V5 tags. Neurons were stimulated twice, first at *T*_stim_ = 5 days and then at *T*_stim_ = 6 days, each time by 55 mM KCl for 1 h. Scale bar, 5 µm. **k**, V5 signal relative change from baseline plotted against recovered time after AAV transduction for the XRIs shown in **j**. Thin lines, traces from individual XRIs; thick line, the averaged trace over all XRIs. **l**, V5 signal relative change from baseline plotted against recovered time after AAV transduction for XRIs in neurons under two sequential KCl stimulations as described in **j** (*n* = 16 neurons from two cultures). Thick centerline, mean; darker boundary in the close vicinity of the thick centerline, s.e.m.; lighter boundary, s.d. *P = 0.0118; **P = 0.0097; Kruskal–Wallis analysis of variance followed by post hoc Dunnʼs tests between the peak V5 signal relative change during *T* = 5–6 (or 6–7) days after AAV transduction and the baseline V5 signal relative change (that is, the V5 signal relative change averaged over *T* = 3–5 days after AAV transduction). See Supplementary Table 3 for details of statistical analysis.

**Extended Data Fig. 7:**
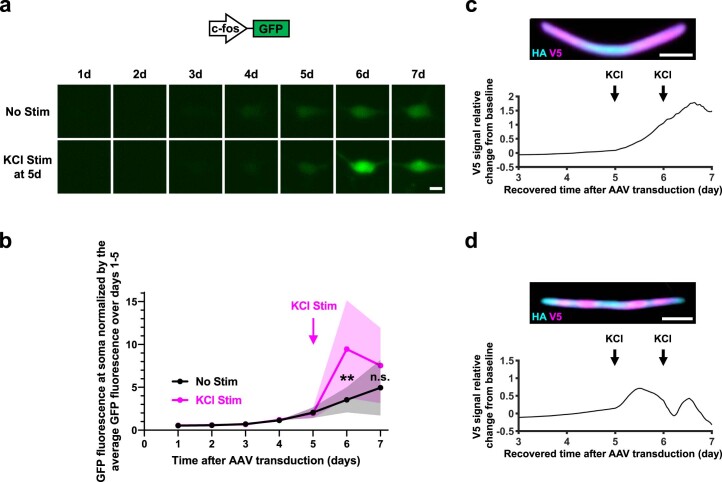
KCl stimulation of cultured neurons with c-fos promoter-driven expression of GFP. (**a**) Construct schematic of GFP under c-fos promoter and representative confocal images of live cultured mouse hippocampal neurons in the GFP channel 1-7 days (1d-7d) after AAV transduction, without (upper row) and with (lower row) 55 mM KCl stimulation for 3 hours on 5d. All images were captured under the same imaging condition. Scale bar, 10 µm. (**b**) GFP fluorescence at soma (normalized by the average GFP fluorescence at soma over days 1-5) versus time (n = 11 neurons from 2 cultures for ‘No Stim’ group; n = 12 neurons from 2 cultures for ‘KCl Stim’ group). n.s., not significant; **, P = 0.0038; two-sided Wilcoxon rank sum tests with Holm-Sidak correction between ‘No Stim’ and ‘KCl Stim’ on day 6 or day 7 after AAV transduction. See Supplementary Table 3 for details of statistical analysis. Centerline, mean; shaded boundary, standard deviation. (**c,d**) Top, confocal images of XRIs (as examples in addition to Fig. 3j) in two cultured mouse hippocampal neurons expressing constructs in Fig. 3a, taken after fixation (7 days after AAV transduction), Nissl staining, and immunostaining against HA and V5 tags; bottom, V5 signal relative change from baseline plotted against recovered time after AAV transduction for the corresponding XRI. Scale bars, 5 µm. Neurons were stimulated twice, first at T_stim_ = 5 days and then at T_stim_ = 6 days, each time by 55 mM KCl for 1 hour.

We next explored whether XRIs could be used to record gene expression timecourse under mammalian immediate early gene (IEG) promoter activation. IEG promoters, such as the c-fos promoter^29^, are used widely to couple the expression of reporter proteins to specific cellular stimuli^30^. By using the c-fos promoter to drive the expression of XRI subunits tagged by a unique epitope tag, here the V5 tag, the timecourse of c-fos-promoter-driven expression could be recorded along the XRI filament, and read out by measuring the intensity profiles of V5 immunostaining signals along the filament. We chose to use the V5 tag here, instead of the previously used FLAG tag, so that each new XRI construct would be tagged by a unique epitope tag: in future usage of XRIs, one may want to coexpress several XRI constructs in the same cell to achieve multiplexed recording of several different kinds of biological signals, readable via multiplexed immunostaining against distinct epitope tags. We expressed HA-bearing XRI, driven by the UBC promoter, in neurons using AAV as in the experiments in Fig. 2, along with the new V5-bearing XRI driven by the c-fos promoter (Fig. 3a–c). We diluted the AAV for the V5-bearing XRI (the final titer was 25% of that of the AAV for the HA-bearing XRI) so that the expression of HA-bearing monomers (and thus the HA portion of the final XRI assembly) would dominate over V5-bearing monomers, and serve as a reliable integral substrate. We stimulated the neurons for 3 h with 55 mM KCl—a common method to induce neuronal depolarization known to result in an increase in c-fos expression^31–33^. As expected, in the KCl-stimulated neurons, we observed low V5 immunofluorescence at the middle of the XRI, and towards both ends of the XRI the V5 immunofluorescence increased, resulting in peak-like patterns on each of the two sides of the XRI, eventually falling off (Fig. 3d,e, right). This peak-like pattern of V5 immunofluorescence was not observed in XRIs in neurons without KCl stimulation (‘No Stim’ group; Fig. 3d,e, left). The HA intensity fluctuated the opposite way of the V5 intensity (Fig. 3d,e, right), as expected because, as discussed earlier, V5-bearing monomers would dilute down the line density of HA-bearing monomers; as long as the new V5 units being added were not competing with HA units being added, but simply were spacing the HA units out further, the line integral of HA units being added would be a useful measure of absolute time, at least over the timescale of this experiment (see above). Using the relationship between time and the line integral of HA intensity obtained above (Fig. 2h), we plotted the relative change of V5 signal from baseline (baseline defined as the V5 signal when the fraction of the line integral of HA intensity was zero) along the XRI versus time. As expected, a peak of V5 signal was observed after the recovered time of day 5, which matched the actual time of KCl stimulation (Fig. 3e, bottom row), while in neurons without KCl stimulation, the V5 signal stayed relatively unchanged. To validate the XRI-recorded timecourse of c-fos-promoter-driven expression, we performed time-lapse imaging, one image per day, of cultured neurons transduced with an AAV construct encoding c-fos-promoter-driven expression of GFP, under the same KCl stimulation (Extended Data Fig. 7a). We found that the waveform of GFP intensity over time was similar to the XRI-recorded timecourse of c-fos-promoter-driven expression (compare Extended Data Fig. 7b with Fig. 3e, bottom row), although the nonstimulated case accumulated a small amount of GFP, presumably because of baseline neural activity in the culture^34^, whereas the XRI case did not exhibit a peak in the nonstimulated case, perhaps because the baseline neural activity provided a constant background level of available XRI subunits.

To assess the sensitivity of the XRI fos recorder, we next performed XRI recording of c-fos-promoter-driven XRI expression with different doses and durations of KCl stimulation (Fig. 3f), analyzing the average poststimulation XRI amplitude, the peak poststimulation XRI amplitude and the rising slope of the XRI after KCl stimulation. We found that the XRI system responded with brighter and higher-slope signals with stronger and longer stimulations, than with weaker and shorter ones (Fig. 3g–i). To gauge whether this sensitivity could be applied to detect sequential neural stimulations, we performed two sequential KCl stimulations of the same neural population, separated by 1 day, and found that we could recover the times of both stimulation events via c-fos-promoter-driven expression of XRI subunits in cultured neurons (Fig. 3j,k (results from a representative neuron), Fig. 3l (averaged results from all neurons) and Extended Data Fig. 7c,d (results from additional neurons)).

**Fig. 4:**
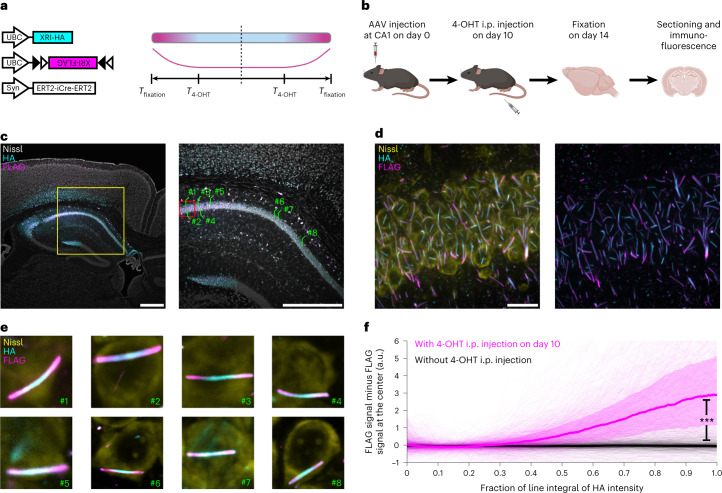
In vivo XRI self-assembly in mouse brain. **a**,**b**, Schematics of the AAV constructs (**a**, left), expected epitope distribution along the XRI protein self-assembly (**a**, right), and experiment pipeline (**b**) in this XRI self-assembly experiment in mouse brain. AAVs were injected into the dorsal CA1 area of the brains of 3-month-old mice on day 0, followed by 4-OHT i.p. injection on day 10 and then fixation via 4% paraformaldehyde perfusion on day 14. The preserved brains were then sectioned at 50 µm coronally and stained with anti-HA, anti-FLAG and Nissl stain. **c**, Confocal images of a representative brain section from the experiment described in **b**. Yellow square in left panel, boundary of the region of interest enlarged in the right panel; red square in right panel, boundary of the region of interest enlarged in **d**; lines and numbers in right panel, locations of the neurons shown in **e**; scale bars, 500 µm. **d**, Maximum intensity projection of a 4.4-µm-thick volume in the region of interest indicated in the red square in the right panel in **c**. Some of the XRIs are not contained completely within the volume for this maximum intensity projection in the Z (depth) dimension and therefore are not fully shown in these two-dimensional images. Scale bar, 20 µm. **e**, Confocal images of representative CA1 neurons indicated in the right panel in **c**. **f**, FLAG signal minus the FLAG signal at the center averaged and plotted against the fraction of the line integral of HA intensity along the XRI. *n* = 893 XRIs from 835 CA1 neurons from one brain section (shown in **c**) from one mouse with 4-OHT i.p. injection on day 10 (magenta) and *n* = 598 XRIs from 475 CA1 neurons from one brain section from one mouse without 4-OHT i.p. injection (black). The line integral of HA intensity was defined as ‘1’ for day 14, the time of fixation and thus the end of XRI growth. Colored lines, median; colored, shaded boundaries, interquartile range; lighter thin lines, data from individual XRIs. ***P < 0.0001; two-sided Wilcoxon rank sum test. See Supplementary Table 3 for details of statistical analysis.

Next, we tested if XRI can preserve temporal information in the living mammalian brain. We took the same XRI AAVs used in Fig. 2 and coinjected them into the hippocampal CA1 region of the brains of wild-type adult mice (Fig. 4a,b). Based on previous experience from us and others^35,36^ on the AAV-mediated gene delivery of Cre (in the experiment here ERT2-iCre-ERT2 was delivered) into the mouse brain in vivo, we doubled the expression time to 14 days for this in vivo experiment, so that 4-OHT was administered into mouse via intraperitoneal (i.p.) injection^37^ at 10 days after AAV injection (five sevenths of the way through the experimental timecourse) to induce the enzymatic activity of ERT2-iCre-ERT2, which triggers the expression of the FLAG-bearing XRI, and then the mouse brain was fixed and sectioned 14 days after AAV injection for downstream immunofluorescence (see experiment pipeline in Fig. 4b). After immunofluorescence imaging of the resulted brain slices, we observed abundant expression of XRI in neurons in the CA1 area (Fig. 4c–e, low-magnification, high-magnification images and close-up images of individual representative neurons, respectively). Similar to what was observed in cultured neurons in Fig. 2, the FLAG immunofluorescence had a strong polarized pattern in the XRIs formed in vivo, confirming that XRI can indeed preserve temporal information in the living mammalian brain.

We analyzed the XRIs in 835 CA1 neurons in confocal imaged volumes and plotted the absolute, baseline subtracted (baseline defined as the signal at the center of XRI) FLAG signals with respect to the fraction of the line integral of HA intensity, and performed the same analysis on XRIs in 475 CA1 neurons in another mouse that underwent the same experimental pipeline but without 4-OHT injection (Fig. 4f). FLAG signals in the mouse without 4-OHT injection were flat with respect to the fraction of the line integral of HA intensity, while those in the mouse with 4-OHT injection on day 10 began to rise when the fraction of the line integral of HA intensity reached 0.3. This 0.3 value alone does not provide absolute information about the time axis, without an in vivo calibration of the timecourse as done in vitro for Fig. 2, but we note that this 0.3 value, from this day 10 4-OHT injection amidst a 14-day in vivo experiment, matched the same value obtained for the day 5 4-OHT treatment in the 7-day experiment in cultured neurons (Fig. 2h). Note that, in both cases, 4-OHT was given at a timepoint five sevenths of the way through the total XRI expression time, suggesting that this timepoint corresponds to 30% of the fraction of the line integral of HA intensity, in several neural preparations. Future work on developing XRI for in vivo use should replicate the calibration experiment of Fig. 2h in the living mouse brain, to precisely calibrate the time axis numerically.

## Discussion

In this work, we proposed and experimentally confirmed that cellular physiological information could be recorded onto intracellular, steadily growing, protein chains made out of fully genetically encoded self-assembling proteins, and then read out via routine immunofluorescence and imaging techniques. By screening existing, human-created self-assembling protein candidates and then performing protein engineering to add an ‘insulator’ component to the promising self-assembling protein candidate 1POK(E239Y) to encourage stable, time-ordered longitudinal growth, we developed what we call an XRI, named by analogy to our earlier signaling reporter island technology (SiRI, which also uses self-assembling peptides, but in that case to create a spatial encoding of indicator identity^38^)—a fully genetically encoded system for recording biological information via self-assembling protein chains. We defined, provided rationale for and validated a calibratable measure of time, the fractional cumulative expression of HA-bearing monomers, to calibrate the time axis onto the information recorded on the XRI via ordered epitope tags. We applied XRIs to record c-fos-promoter-driven gene expression in cultured mouse hippocampal neurons after depolarization, and applied the fractional cumulative expression of HA-bearing monomers to recover the time axis and c-fos-promoter-driven gene expression solely from information read out from XRI via immunostaining and imaging. We showed that XRI could preserve the temporal order of protein monomers expressed in the living mouse brain. Thus, XRIs function in several biological systems, including the live mammalian brain, in encoding cellular physiological signals into a linear, optically readable protein chain.

Compared to nucleic acid-based systems, which require nucleic acid sequencing methods that require dissociation and/or lysis of cells^7–20^, reading out recorded information from a protein-based system through imaging only requires routine immunofluorescence techniques and conventional microscopes, available to many biology groups already, without the need for additional hardware investment. Such preservation of cellular physiological information within the native environment offered by our protein-based system also would enable correlation of the recorded biological information with other kinds of structural and molecular information associated with the cellular population, such as the spatial location, cell type and presence of protein and other markers in the recorded cells^5,6,37^, some of which may be causally involved with the creation of the physiological signals, or that result from the physiological signals. Such kinds of multimodal data could enable the analysis of how specific cellular machinery drive, or result from, complex timecourses of physiological stimuli. For example, by offering the ability to record gene expression timecourse in single cells, as shown here, the proposed protein-based XRI system will enable the study of gene expression timecourse as a result of specific cellular inputs and/or drug treatments^39,40^. This could be useful, amongst many other possibilities, for the investigation of circadian gene rhythms^41^ and rhythms of other genes that change in complex ways over time. XRIs could be used to record transcription factor activities^42^, as an information storage platform to externally introduce unique cellular barcodes into single cells for cell identification^43^, or to investigate transcription dynamics by integration with single-cell RNA-seq^44,45^, as just a few out of many possibilities.

The XRI system has some notable limitations, currently. The first limitation is that it is not yet ready for applications in dividing cells. This might make sense—we are creating a macroscopic protein assembly without its own ability to copy and be sorted into daughter cells, and so it would stand to reason that it would do poorly in dividing cells: either the XRI would stay intact, meaning that one daughter cell would be XRI-free, or the XRI would be split, meaning a loss of information; in either case, XRI functionality is compromised. The second limitation is the requirement of a time calibration experiment to establish the time axis. Our current method, which relies on the synchronization of XRI growth across cells, is sufficient to enable recording at the time resolution of around 1 day. Future research could equip an XRI with its own clock, for example, by using light-inducible^46^ or chemical-inducible^47^ subunits, which respond to an external stimulus at a defined time, to encode that time on the protein chain. In this way users could write markers of specific times along the XRI chain at defined time points independent of the growth kinetics, so that no calibration is needed. Finally, in this study, we did not explore the upper limit of recording duration of the XRI system, instead focusing on a range where XRIs operated safely and efficaciously. Further work may try to maximize the duration of recording XRIs can support, potentially requiring additional protein engineering.

Future work may include the development of mechanisms for coupling XRI expression to other biological dynamics and processes, which would substantially broaden the kinds of biological information XRI could record. For example, the c-fos promoter we used in the study is a natural ‘tool’ that couples c-fos promoter activity to XRI expression. Ongoing activities to engineer promoters and expression systems that respond to calcium^48,49^ and other physiological dynamics^30,50^ would enable XRI recordings of these dynamics. Another future direction will be to expand the XRI system for multiplexed recording of more kinds of biological information onto the same polymer chain, using additional epitope tags for each kind of biological information, and more multiplexed immunostaining methods^38^ to read out each information. For example, one could use tags A and B to encode the gene expression history of genes 1 and 2, respectively, and use tag C to encode the calcium signal, by expressing all the components simultaneously, and then immunostaining all the tags after fixation. Future work may also improve XRI designs to reach time resolutions of recording well below roughly 1 day, perhaps even towards minute timescales or better, while still allowing a recording duration over several days or even more.

## Methods

### Animals and neuron cultures

All procedures involving animals at the Massachusetts Institute of Technology were conducted in accordance with the United States National Institutes of Health Guide for the Care and Use of Laboratory Animals and approved by the Massachusetts Institute of Technology Committee on Animal Care and Biosafety Committee.

For Figs. 1, 2 and 3 and Extended Data Figs. 1, 2 and 4–7, hippocampal neurons were prepared from postnatal day 0 or 1 Swiss Webster mice (Taconic) (both male and female mice were used) as previously described^51^ with the following modifications: dissected hippocampal tissue was digested with 50 U papain (Worthington Biochem) for 6–8 min, and digestion was then stopped with ovomucoid trypsin inhibitor (Worthington Biochem). Cells were plated at a density of 20,000–30,000 per glass coverslip coated with diluted Matrigel in a 24-well plate. Cells were seeded in 100 μl neuron culture medium containing Minimum Essential Medium (MEM, no glutamine, no phenol red; Gibco), glucose (25 mM, Sigma), holo-Transferrin bovine (100 µg ml^–1^, Sigma), HEPES (10 mM, Sigma), glutaGRO (2 mM, Corning), insulin (25 µg ml^–1^, Sigma), B27 supplement (1×, Gibco), and heat-inactivated fetal bovine serum (10% in volume, Corning), with final pH adjusted to 7.3–7.4 using NaOH. After cell adhesion, additional neuron culture medium was added. AraC (2 µM, Sigma) was added at 2 days in vitro (DIV 2), when glial density was 50–70% of confluence. Neurons were grown at 37 °C and 5% CO_2_ in a humidified atmosphere in a neuron incubator, with 2 ml total medium volume in each well of the 24-well plate.

### Molecular cloning

The DNAs encoding the protein motifs used in this study were mammalian-codon optimized and synthesized by Epoch Life Science and then cloned into mammalian expression backbones, pAAV-UBC (for constitutive expression), pAAV-UBC-FLEX (for Cre-dependent expression) or pAAV-cFos (for expression driven by the c-fos promoter) for DNA transfection in cultured neurons and AAV production by Janelia Viral Tools. See Supplementary Table 1 for sequences of the motifs and Supplementary Table 2 for all tested constructs.

### DNA transfection and AAV transduction in cultured neurons

For Fig. 1d,g and Extended Data Fig. 1a,b, cultured neurons were transfected at DIV 7 with a commercial calcium phosphate transfection kit (Invitrogen) as previously described^52^. Briefly, for transfection in each coverslip/well in the 24-well plate, 5–50 ng total XRI plasmid (5–25 ng of each plasmid when cotransfecting several plasmids), 200 ng pAAV-Syn-ERT2-iCre-ERT2 plasmid (only added in neurons for 4-OHT induction experiments), and pUC19 plasmid as a ‘dummy’ DNA plasmid to bring the total amount of DNA to 1,500 ng (to avoid variation in DNA-calcium phosphate coprecipitate formation) were used. The cells were washed with acidic MEM buffer (containing 15 mM HEPES with final pH 6.7–6.8 adjusted with acetic acid (Millipore Sigma)) after 45–60 min of calcium phosphate precipitate incubation to remove residual precipitates.

For Figs. 1h–j, 2 and 3 and Extended Data Figs. 1c–h, 2, 4, 6 and 7, cultured neurons were transduced at DIV 7 with AAVs (except for AAV9-Syn-ERT2-iCre-ERT2, which was added at DIV 4) by adding the concentrated AAV stocks (serotype AAV9; Janelia Viral Tools) into neuron culture medium at the following final concentrations in 2 ml neuron culture medium per well: for 4-OHT induction experiments, AAV9-UBC-XRI-HA at 5.56 × 10^9^ GC ml^–1^, AAV9-UBC-FLEX-XRI-FLAG at 1.88 × 10^10^ GC ml^–1^ and AAV9-Syn-ERT2-iCre-ERT2 at 8.60 × 10^9^ GC ml^–1^; for timecourse recording experiments of c-fos-promoter-driven expression, AAV9-UBC-XRI-HA at 5.56 × 10^9^ GC ml^–1^ and AAV9-cFos-XRI-V5 at 1.39 × 10^9^ GC ml^–1^; for XRI live cell imaging and electrophysiology experiments, AAV9-UBC-mEGFP-P2A-XRI-HA at 2.78 × 10^10^ GC ml^–1^.

### Chemical treatments and stimulations of cultured neurons

For 4-OHT treatments in Figs. 1 and 2 and Extended Data Figs. 2–4, the original culture medium of neuron cultures was transferred into a fresh 24-well plate, where the medium from different neuron cultures were stored in different wells, and kept in the neuron incubator until the end of 4-OHT treatment; 2 ml fresh neuron culture medium containing 1 µM 4-OHT (Sigma, catalog no. H6278) was added into each well of neuron culture. The neuron cultures were then placed back to the neuron incubator and incubated for 15 min, followed by a brief wash in MEM medium. Finally, the MEM medium was removed and the original neuron culture medimu was transferred back to the corresponding wells of neuron culture. The neuron cultures were then placed back in the neuron incubator.

For KCl treatments in Fig. 3 and Extended Data Fig. 7, KCl depolarization solution was prepared, which contained 170 mM KCl, 2 mM CaCl_2_, 1 mM MgCl_2_ and 10 mM HEPES. Then, KCl depolarization medium was prepared by mixing KCl depolarization solution and fresh neuron culture medium, so that the final concentration of K^+^ after mixing was 55 mM or 20 mM (taking into account the K^+^ from the fresh neuron culture medium). The original culture medium of neuron cultures was transferred into a fresh 24-well plate, where the medium from different neuron cultures were stored in different wells, and kept in the neuron incubator until the end of the KCl-induced depolarization treatment; 2 ml KCl depolarization medium was added to each well of neuron culture. Neuron cultures were then placed back into the neuron incubator and incubated for 10 min, 30 min, 1 h or 3 h. Finally, the KCl depolarization medium was removed and the original neuron culture medium was transferred back into the corresponding wells of the neuron cultures. The neuron cultures were then placed back in the neuron incubator.

### DNA transfection in cultured U2OS cells

Human bone osteosarcoma epithelial cells (U2OS cells; ATCC) were maintained between 10% and 90% confluence at 37 °C with 5% CO_2_ in DMEM (Gibco) with the addition of 10% heat-inactivated fetal bovine serum (HI-FBS) (Corning), 1% penicillin–streptomycin (Gibco) and 1% sodium pyruvate (Gibco), in glass-bottom 24-well plates pretreated with 75 μl diluted Matrigel (250 μl Matrigel (Corning) diluted in 12 ml DMEM) per well at 37 °C for 30–60 min. The DNA plasmid was transiently transfected into U2OS cells using the TransIT-X2 Dynamic Delivery System kit (Mirus Bio) according to the manufacturer’s protocol.

### Electrophysiology

For Extended Data Fig. 2a–e, whole-cell patch clamp recordings were performed using Axopatch 200B or Multiclamp 700B amplifiers, a Digidata 1440 digitizer and a personal computer running pClamp (Molecular Devices). Cultured neurons were patched on DIV 14–16 (7–9 days after AAV transduction). Neurons were bathed in room temperature Tyrode solution containing 125 mM NaCl, 2 mM KCl, 3 mM CaCl_2_, 1 mM MgCl_2_, 10 mM HEPES, 30 mM glucose and the synaptic blockers 0.01 mM NBQX and 0.01 mM GABAzine. The pH of the Tyrode solution was adjusted to 7.3 with NaOH and the osmolarity was adjusted to 300 mOsm with sucrose. Borosilicate glass pipette (Warner Instruments) with an outer diameter of 1.2 mm and a wall thickness of 0.255 mm was pulled to a resistance of 5–10 MΩ with a P-97 Flaming/Brown micropipette puller (Sutter Instruments) and filled with a pipette solution containing 155 mM K-gluconate, 8 mM NaCl, 0.1 mM CaCl_2_, 0.6 mM MgCl_2_, 10 mM HEPES, 4 mM Mg-ATP and 0.4 mM Na-GTP. The pH of the pipette solution was adjusted to 7.3 with KOH and the osmolarity was adjusted to 298 mOsm with sucrose.

### Animals and mouse surgery

All procedures involving animals at Boston University were conducted in accordance with the United States National Institutes of Health Guide for the Care and Use of Laboratory Animals and approved by the Boston University Institutional Animal Care and Use and Biosafety Committees.

For experiments in Fig. 4 and Extended Data Figs. 1j,k and 3, all surgeries were performed under stereotaxic guidance, and coordinates were given relative to bregma (in millimeters). Dorsal ventral injections were calculated and zeroed out relative to the skull. Wild-type C57BL/6 mice (3 months of age; male; Charles River Laboratories) were placed into a stereotaxic frame (Kopf Instruments) and anesthetized with 3% isoflurane during induction (lowered to 1–2% to maintain anesthesia throughout surgery). Ophthalmic ointment was applied to both eyes. Hair was removed with a hair removal cream and the surgical site was cleaned with ethanol and betadine. Following this, an incision was made to expose the skull. Bilateral craniotomies involved drilling windows through the skull above the injection site using a 0.5 mm diameter drill bit. Coordinates were −2.0 anteroposterior (AP), ±1.5 mediolateral (ML) and −1.5 dorsoventral (DV) for dorsal CA1.

For experiments in Fig. 4, the AAV mixture for injection was prepared by mixing the AAV stocks (serotype AAV9; Janelia Viral Tools) at the following final concentrations: AAV9-UBC-XRI-HA at 1.48 × 10^13^ GC ml^–1^, AAV9-UBC-FLEX-XRI-FLAG at 3.77 × 10^13^ GC ml^–1^ and AAV9-Syn-ERT2-iCre-ERT2 at 1.72 × 10^13^ GC ml^–1^. For experiments in Extended Data Figs. 1j,k and 3, the following AAV concentrations were used for injection (serotype AAV9; Janelia Viral Tools): AAV9-Syn-GFP at 5.75 × 10^13^ GC ml^–1^; AAV9-UBC-XRI-HA at 1.00 × 10^13^ GC ml^–1^. Mice were injected with 0.6–1.0 μl of the AAV mixture at the target site using a mineral oil-filled 33-gauge beveled needle attached to a 10 μl Hamilton microsyringe (701LT; Hamilton) in a microsyringe pump (UMP3; WPI). The needle remained at the target site for 5 min postinjection before removal. Mice received buprenorphine i.p. following surgery and were placed on a heating pad during surgery and recovery.

### 4-OHT injection in mice

For experiments in Fig. 4, 4-OHT (Sigma) was dissolved in 100% ethanol (Sigma) at 100 mg ml^–1^ by vortexing for 5 min. Next, the solution was mixed with corn oil (Sigma) to obtain a final concentration of 10 mg ml^–1^ 4-OHT by vortexing for 5 min and then sonicating for 30–60 min until the solution was clear. The 10 mg ml^–1^ 4-OHT solution was then loaded into syringes and administered to mice via i.p. injection at 40 mg kg^–1^.

### Histology

For experiments in Fig. 4 and Extended Data Figs. 1j,k and 3, mice were perfused transcardially with 1× PBS followed by 4% paraformaldehyde in 1× PBS. The brain was gently extracted from the skull and postfixed in 4% paraformaldehyde in 1× PBS overnight at 4 °C. The brain was then incubated in 100 mM glycine in 1× PBS for 1 h at RT, and then the brain was transferred into 1× PBS and stored at 4 °C until slicing. The brain was sliced to 50-µm thickness coronally using a vibratome (Leica), and then stored in 1× PBS at 4 °C until immunofluorescence staining.

### Immunofluorescence

#### Immunofluorescence of cultured cells

Fof Figs. 1–3 and Extended Data Figs. 1, 4–6 and 7c,d, cells were fixed in TissuePrep-buffered 10% formalin for 10 min at room temperature (RT) followed by three washes in 1× PBS, 5 min each at RT. Cells were then incubated in MAXBlock blocking medium (Active Motif) supplemented with final concentrations of 0.1% Triton X-100 and 100 mM glycine for 20 min at RT, followed by three washes in MAXwash washing medium (Active Motif), 5 min each at RT. Next, cells were incubated with primary antibodies in MAXbind staining medium (Active Motif) overnight at 4 °C, followed by three washes in MAXwash washing medium, 5 min each at RT. Cells were then incubated with fluorescently labeled secondary antibodies and NeuroTrace Blue Fluorescent Nissl Stain (Invitrogen) in MAXbind staining medium overnight at 4 °C, followed by three washes in MAXwash washing medium, 5 min each at RT. The cells were then stored in 1× PBS at 4 °C until imaging.

#### Immunofluorescence of brain slices

For Fig. 4 and Extended Data Figs. 1 and 3, brain slices were blocked overnight at 4 °C in MAXBlock blocking medium, followed by four washes for 30 min each at RT in MAXWash washing medium. Next, slices were incubated with primary antibodies in MAXbind staining medium overnight at 4 °C, and then washed in MAXWash washing medium four times for 30 min each at RT. Next, slices were incubated with fluorescently labeled secondary antibodies and NeuroTrace Blue Fluorescent Nissl Stain (Invitrogen) in MAXbind staining medium overnight at 4 °C, and then washed in MAXWash washing medium four times for 15 min each at RT. The slices were then stored in 1× PBS at 4 °C until imaging.

#### Expansion microscopy of cultured cells

For Extended Data Fig. 6, cell cultures on round coverslips were fixed in 4% paraformaldehyde (Electron Microscopy Sciences) and 0.1 % glutaraldehyde (Electron Microscopy Sciences) in 1× PBS for 10 min at RT. Cells were then incubated in 0.1 % sodium borohydride (Sigma) in 1× PBS for 7 min and then in 100 mM glycine (Sigma) in 1× PBS for 10 min, both at RT.

Acryloyl-X (6-((acryloyl)amino)hexanoic acid, succinimidyl ester (AcX) (Invitrogen) was resuspended in anhydrous dimethylsulfoxide (Invitrogen) at a concentration of 10 mg ml^–1^ and stored in a desiccated environment at −20 °C. For anchoring, cells were incubated in 200 μl AcX at a concentration of 0.1 mg ml^–1^ in a 2-(N-morpholino)ethanesulfonic acid (MES)-based saline (100 mM MES, 150 mM NaCl) overnight at 4 °C. Then, cells were washed with 1× PBS three times at RT for 5 min each.

Gelation solution containing 1.1 M sodium acrylate (Sigma), 2 M acrylamide (Sigma), 90 ppm *N*,*N*′-methylenebisacrylamide (Sigma), 1.5 ppt ammonium persulfate (Sigma) and 1.5 ppt tetramethylethylenediamine (Sigma) in 1× PBS was prepared fresh. Cells were first incubated on ice for 10 min with shaking to prevent premature gelation and enable diffusion of solution into samples. A gelation chamber was prepared by placing two No. 1.5 coverslips on a glass slide spaced by about 8 mm to function as insulators on either end of the neuronal coverslip to avoid compression and each coverslip containing a neuronal cell culture sample was placed on a gelation chamber with the cells facing down. The gelation chamber was filled with gelation solution and a coverslip placed over the sample and across the two insulators to ensure the sample was covered with gelling solution and no air bubbles were formed on the sample. Samples were incubated at 37 °C for 1 h in a humidified atmosphere to complete gelation. Following gelation, the top coverslip was removed from the samples, and only the sample gel was transferred into a 1.5 ml tube containing 1 ml denaturation buffer, consisting of 5% (w/v) sodium dodecyl sulfate (SDS), 200 mM NaCl and 50 mM Tris at pH 8. Gels were incubated in denaturation buffer overnight at RT and then 3 h at 80 °C, followed by washing in water overnight at RT to remove residual SDS. Gels were then stored in 1× PBS at 4 °C before immunostaining.

For immunostaining and imaging, gels were first incubated in bovine serum albumin (BSA) blocking solution that contains 1% BSA, 0.5% Triton X in 1× PBS for 1 h at RT then with primary antibodies in MAXbind staining medium overnight at 4 °C. Gels were washed three times in BSA blocking solution for 30 min each at RT and incubated with fluorescently labeled secondary antibodies in MAXbind Staining Medium overnight at 4 °C. Gels were then washed three times in BSA blocking solution for 30 min each at RT and expanded in water overnight at 4 °C before imaging.

#### Antibodies and Nissl stain

Primary antibodies (unless specified below, 1:500 for immunofluorescence of cultured cells, 1:250 for immunofluorescence of brain slices and 1:200 for expansion microscopy of cultured cells): anti-HA (Santa Cruz, catalog no. sc-7392), anti-FLAG (Invitrogen, catalog no. 740001), anti-V5 (Abcam, catalog no. ab9113), anti-NeuN (Synaptic Systems, catalog no. 266004, 1:1,000 for brain slices), anti-GFAP (Cell Signaling Technology, catalog no. 12389, 1:500 for brain slices), anti-Iba1 (Wako Chemicals, catalog no. 019-19741, 1:500 for brain slices), anti-Synaptophysin (Sigma, catalog no. S5768, 1:500 for brain slices), anti-Cleaved Caspase-3 (Cell Signaling Technology, catalog no. 9664, 1:250 for brain slices), anti-γH2AX (Millipore, catalog no. 05-636, 1:500 for brain slices), anti-Hsp70 (Cell Signaling Technology, catalog no. 4872, 1:200 for brain slices), anti-Hsp27 (Cell Signaling Technology, catalog no. 2402, 1:50 for brain slices). Fluorescent secondary antibodies (unless specified below, 1:500 for immunofluorescence of cultured cells, 1:500 for immunofluorescence of brain slices and 1:200 for expansion microscopy of cultured cells): Goat anti-Mouse IgG2a Alexa Fluor 647 (Invitrogen, catalog no. A-21241), Goat anti-Mouse IgG2a Alexa Fluor 546 (Invitrogen, catalog no. A-21133), Goat anti-Chicken IgY Alexa Fluor Plus 647 (Invitrogen, catalog no. A-32933), Goat anti-Rabbit IgG Alexa Fluor Plus 647 (Invitrogen, catalog no. A-32733, 1:200 in Extended Data Fig. 3), Goat anti-Rabbit IgG Alexa Fluor 546 (Invitrogen, catalog no. A-11035), Goat anti-Guinea Pig IgG Alexa Fluor 488 (Invitrogen, catalog no. A-11073), Goat anti-Guinea Pig IgG Alexa Fluor 647 (Invitrogen, catalog no. A-21450, 1:200 in Extended Data Fig. 3), Goat anti-Mouse IgG2a Alexa Fluor 546 (Invitrogen, catalog no. A-21133, 1:200 in Extended Data Fig. 3), Goat anti-Mouse IgG1 Alexa Fluor 546 (Invitrogen, catalog no. A-21123, 1:200 in Extended Data Fig. 3) and Donkey Anti-Rabbit IgG CF543 (Biotium, catalog no. 20308). Nissl stain: NeuroTrace Blue Fluorescent Nissl Stain (Invitrogen, catalog no. N21479, 1:500 for immunofluorescence of cultured cells and 1:250 for immunofluorescence of brain slices).

### Fluorescence microscopy of live cells and immunostained samples

Fluorescence microscopy was performed on a spinning disk confocal microscope (Yokogawa CSU-W1 Confocal Scanner Unit on a Nikon Eclipse Ti microscope) equipped with a ×40 1.15 numerical aperture water immersion objective (Nikon MRD77410), a ×10 objective, a Zyla PLUS 4.2 Megapixel camera controlled by NIS-Elements AR software, and laser/filter sets for 405 nm, 488 nm, 561 nm and 640 nm optical channels. For each field of view under the ×40 objective, multichannel volumetric imaging was performed at 0.4 µm per Z step. Imaging parameters were kept the same for all samples within a set of experiments (for example, a set of 4-OHT induction experiments in which samples were treated with 4-OHT at different time points).

### RNA-sequencing

For Extended Data Fig. 2f–h, RNA was extracted from individual neuron cultures in 24-well plates with Trizol (Thermo Fisher) and purified with an RNeasy Mini Kit (Qiagen). RNA quality was confirmed using a Femto Pulse system (Agilent). cDNA was generated from 2 ng total RNA using the SMART-Seq v.4 Ultra Low Input RNA Kit (Takara Bio) amplifying for ten cycles and confirmed using a Fragment Analyzer (Agilent). Amplified cDNA (200 ng) was prepared for Illumina sequencing by Nextera Flex (Illumina) using half volume reactions with six cycles of amplification. Final libraries were quantified on the Fragment Analyzer and by qPCR on a LC480 Light Cycler (Roche). Libraries were sequenced on a MiSeq (Illumina) using 75 nucleotide (nt) paired end reads. Sequences were mapped to GRCm38 (mm10) reference genome (with gene annotations obtained from Ensembl). Gene expression raw counts were assessed by RSEM and then were normalized and batch-effect adjusted using DESeq2 (ref. ^53^), followed by differential expression analysis and statistics using DESeq2.

### Image analysis

Image analysis was performed in ImageJ (ImageJ National Institutes of Health) and MATLAB (MathWorks).

#### Intensity profile measurements

First, the somata of neurons in the images were identified by the Nissl staining (in samples without ExM) or the anti-NeuN staining (in samples with ExM) channel, and XRI(s) in the soma of each neuron were identified by the anti-HA channel. If several XRIs were present in a soma, the XRI with the longest length as well as any XRI with length above half of that longest length was selected for downstream analysis. For each XRI, a curved centerline was drawn along the longitudinal direction of XRI in the anti-HA channel. The centerline width was set to half of the width of the XRI. The intensity profiles along this centerline were measured in the anti-HA channel (and called the HA line profile) and in other XRI epitope staining channels, such as in the anti-FLAG channel (called the FLAG line profile) or anti-V5 channel (called the V5 line profile).

#### Readout information from intensity profiles

See Extended Data Fig. 5 for the process flow of extracting information from the intensity profiles of XRIs. Each of the HA, FLAG or V5 line profiles was split into two half line profiles using the geometric center point of the XRI (50% length point along the centerline, measuring from the end of the XRI) as the ‘split point’. Each of the half HA line profiles (*H*) was then converted into a line integrals of HA (*H*_integral) for every position (*p*) along the half XRI, by integrating the line profile with respect to the distance (*d*) along the half centerline starting from the split point (where *d* = 0):

$$H\_\mathrm{integral}\left( p \right) = \mathop {\sum}\limits_{d = 0}^p H (d) \cdot \Delta d$$

Then these line integrals of HA were normalized to the maximum integral value (integral from the split point (*d* = 0) to the end of XRI (*d* = End)) so that each line integral of HA started at the value 0 at the geometric center point of the XRI, and increased gradually to the value 1 at the end of the XRI. We define this quantity as the ‘fraction of HA intensity line integral (*H*_ fraction_integral)’:

$$H\_{\mathrm{fraction}}\_{\mathrm{integral}}\left( p \right) = \mathop {\sum}\limits_{d = 0}^p H (d) \cdot \Delta d/\mathop {\sum}\limits_{d = 0}^{\mathrm{End}} H (d) \cdot \Delta d$$

For the corresponding half FLAG (or V5) line profiles (*F*), line integrals (*F*_integral) were also calculated but not normalized:

$$F\_\mathrm{integral}\left( p \right) = \mathop {\sum}\limits_{d = 0}^p F (d) \cdot \Delta d$$

At this point, we have the line integrals of HA and FLAG (or V5), which correspond to the cumulative HA and FLAG (or V5) intensities along each half of the XRI. We then converted the line integrals of FLAG (or V5) line profiles from the position axis (*p*) into the axis of the fraction of HA intensity line integral (*H_* fraction_integral) via variable substitution from *p* to *H*_ fraction_integral (*p*):

$$F\_\mathrm{integral}\left( p \right)\mathop{\longrightarrow}\limits^{{\mathrm{variable}}\,{\rm{substitution}}}F\_\mathrm{integral}\left( {H\_\mathrm{fraction}\_{\rm{integral}}} \right)$$

The FLAG (or V5) intensity change per unit change in the cumulative HA intensity, defined as the FLAG (or V5) signal (*F*_signal), was calculated by taking the derivative of the line integral of FLAG (or V5) with respect to the fraction of HA intensity line integral:

$$F\_\mathrm{signal}\left( {H\_\mathrm{fraction}\_{\rm{integral}}} \right) = \frac{{\Delta F\_\mathrm{integral}\left( {H\_\mathrm{fraction}\_{\rm{integral}}} \right)}}{{\Delta H\_\mathrm{fraction}\_{\rm{integral}}}}$$

At this stage, we obtained the line integral of HA (*H*_ fraction_integral) and the FLAG (or V5) signal (*F*_signal) from each of the halves of the XRI. Next, we searched for an optimal split point near the geometric center of the XRI (searching range was the geometric center ±25% of the total XRI length), so that using this optimal split point, instead of the geometric center, as the split point results in the least difference (in sum of squared differences) between the two FLAG (or V5) signals from the two halves of the splitted XRI. The final extracted FLAG (or V5) signal from this XRI was defined as the point-by-point average of the two FLAG (or V5) signals from the two halves of the XRI splitted using the optimal split point.

#### Calculation of the fraction of HA line integral when FLAG signal begins to rise

The FLAG signal minus the FLAG signal at the center of XRI (that is, the optimal split point as defined above) was plotted against the fraction of HA line integral. The initial rising phase of the FLAG signal (defined as the portion of the FLAG signal between 10% to 50% of the peak FLAG signal) was fitted as a linear function, which was then extrapolated onto the axis of the fraction of HA line integral. The intersection point at the axis of the fraction of the HA line integral was defined as the fraction of HA line integral when the FLAG signal began to rise.

### Statistical analysis

All statistical analysis was performed using the built-in statistical analysis tools in Prism (GraphPad) or MATLAB, except for the statistical analysis of the RNA-seq data, which was performed using DESeq2 in R (The R Foundation). The statistical details of each statistical analysis can be found in the figure legends and in Supplementary Table 3, except for the statistical details of the RNA-seq analysis, which can be found in the figure legends and in Supplementary Table 4.

### Reporting summary

Further information on research design is available in the Nature Portfolio Reporting Summary linked to this article.

## Online content

Any methods, additional references, Nature Portfolio reporting summaries, source data, extended data, supplementary information, acknowledgements, peer review information; details of author contributions and competing interests; and statements of data and code availability are available at 10.1038/s41587-022-01586-7.

## Supplementary information

Supplementary Information Supplementary Tables 1 and 2.

Reporting Summary

Supplementary Table 3 Statistical analysis.

Supplementary Table 4 RNA-seq differential expression analysis. RNA-seq differential expression analysis across mouse genes using DESeq2, comparing neuron cultures expressing GFP via AAV transduction (the ‘GFP’ group; *n* = 14 neuron cultures) with neuron cultures without AAV transduction (the ‘Neg’ group; *n* = 17 neuron cultures) as the baseline group, neuron cultures expressing XRI via AAV transduction (the ‘XRI’ group; *n* = 24 neuron cultures) with the ‘Neg’ group as the baseline group, or the ‘XRI’ group with the ‘GFP’ group as the baseline group. *P* value for the null hypothesis that there is no differential expression across the two groups was from two-sided Wald test with Benjamini–Hochberg correction.

## Data Availability

The sequences of XRIs reported in this paper are available at GenBank (accession numbers: OK539810, OK539811 and OK539812). Plasmids generated in this study and their sequences are available at Addgene (plasmid nos. 178056-178060). The GRCm38 (mm10) reference genome is available at GenBank (accession number: GCA_000001635.2; https://www.ncbi.nlm.nih.gov/data-hub/genome/GCF_000001635.20/) and the corresponding gene annotations are available at Ensembl (release 88; https://ftp.ensembl.org/pub/release-88/gtf/mus_musculus/Mus_musculus.GRCm38.88.gtf.gz). The datasets generated and analyzed in this study are available at Zenodo (record 7130256; https://zenodo.org/record/7130256).

## Code availability

The codes used for data analysis in this study are available at Zenodo (record 7130256; https://zenodo.org/record/7130256).

## References

[1] Greenwald EC, Mehta S, Zhang J (2018). Genetically encoded fluorescent biosensors illuminate the spatiotemporal regulation of signaling networks. Chem. Rev..

[2] Murray E (2015). Simple, scalable proteomic imaging for high-dimensional profiling of intact systems. Cell.

[3] Ragan T (2012). Serial two-photon tomography for automated ex vivo mouse brain imaging. Nat. Methods.

[4] Gao R (2019). Cortical column and whole-brain imaging with molecular contrast and nanoscale resolution. Science.

[5] Lin D (2011). Functional identification of an aggression locus in the mouse hypothalamus. Nature.

[6] S C, MJ V, M G, T H (1989). Expression of c-Fos immunoreactivity in transmitter-characterized neurons after stress. Proc. Natl Acad. Sci. USA.

[7] Kording KP (2011). Of toasters and molecular ticker tapes. PLoS Comput. Biol..

[8] Perli SD, Cui CH, Lu TK (2016). Continuous genetic recording with self-targeting CRISPR–Cas in human cells. Science.

[9] Rodriques SG (2020). RNA timestamps identify the age of single molecules in RNA sequencing. Nat. Biotechnol..

[10] Farzadfard F, Lu TK (2014). Genomically encoded analog memory with precise in vivo DNA writing in living cell populations. Science.

[11] Farzadfard F, Lu TK (2018). Emerging applications for DNA writers and molecular recorders. Science.

[12] Farzadfard F (2019). Single-nucleotide-resolution computing and memory in living cells. Mol. Cell.

[13] Sheth RU, Yim SS, Wu FL, Wang HH (2017). Multiplex recording of cellular events over time on CRISPR biological tape. Science.

[14] Chan MM (2019). Molecular recording of mammalian embryogenesis. Nature.

[15] Tang W, Liu DR (2018). Rewritable multi-event analog recording in bacterial and mammalian cells. Science.

[16] KL F (2017). Synthetic recording and in situ readout of lineage information in single cells. Nature.

[17] Bowling S (2020). An engineered CRISPR-Cas9 mouse line for simultaneous readout of lineage histories and gene expression profiles in single cells. Cell.

[18] Park J (2021). Recording of elapsed time and temporal information about biological events using Cas9. Cell.

[19] Quinn JJ (2021). Single-cell lineages reveal the rates, routes, and drivers of metastasis in cancer xenografts. Science.

[20] Yang D (2022). Lineage tracing reveals the phylodynamics, plasticity, and paths of tumor evolution. Cell.

[21] Garcia-Seisdedos H, Empereur-Mot C, Elad N, Levy ED (2017). Proteins evolve on the edge of supramolecular self-assembly. Nature.

[22] Shen H (2018). De novo design of self-assembling helical protein filaments. Science.

[23] Cranfill PJ (2016). Quantitative assessment of fluorescent proteins. Nat. Methods.

[24] Reuten R (2016). Maltose-binding protein (MBP), a secretion-enhancing tag for mammalian protein expression systems. PLoS ONE.

[25] Kapust RB, Waugh DS (1999). *Escherichia coli* maltose-binding protein is uncommonly effective at promoting the solubility of polypeptides to which it is fused. Protein Sci..

[26] Matsuda T, Cepko CL (2007). Controlled expression of transgenes introduced by in vivo electroporation. Proc. Natl Acad. Sci. USA.

[27] Kim JH (2011). High cleavage efficiency of a 2A peptide derived from porcine teschovirus-1 in human cell lines, zebrafish and mice. PLoS ONE.

[28] Chen F, Tillberg PW, Boyden ES (2015). Expansion microscopy. Science.

[29] Roy DS (2016). Memory retrieval by activating engram cells in mouse models of early Alzheimer’s disease. Nature.

[30] Kawashima T, Okuno H, Bito H (2014). A new era for functional labeling of neurons: activity-dependent promoters have come of age. Front. Neural Circuits.

[31] Malik AN (2014). Genome-wide identification and characterization of functional neuronal activity-dependent enhancers. Nat. Neurosci..

[32] Tyssowski KM (2018). Different neuronal activity patterns induce different gene expression programs. Neuron.

[33] Joo J-Y, Schaukowitch K, Farbiak L, Kilaru G, Kim T-K (2015). Stimulus-specific combinatorial functionality of neuronal c-fos enhancers. Nat. Neurosci..

[34] Rodríguez-Berdini L (2020). The moonlighting protein c-Fos activates lipid synthesis in neurons, an activity that is critical for cellular differentiation and cortical development. J. Biol. Chem..

[35] Zincarelli C, Soltys S, Rengo G, Rabinowitz JE (2008). Analysis of AAV serotypes 1–9 mediated gene expression and tropism in mice after systemic injection. Mol. Ther..

[36] Kaspar BK (2002). Adeno-associated virus effectively mediates conditional gene modification in the brain. Proc. Natl Acad. Sci. USA.

[37] Guenthner CJ, Miyamichi K, Yang HH, Heller HC, Luo L (2013). Permanent genetic access to transiently active neurons via TRAP: Targeted Recombination in Active Populations. Neuron.

[38] Linghu C (2020). Spatial multiplexing of fluorescent reporters for imaging signaling network dynamics. Cell.

[39] Strober BJ (2019). Dynamic genetic regulation of gene expression during cellular differentiation. Science.

[40] Gallo FT, Katche C, Morici JF, Medina JH, Weisstaub NV (2018). Immediate early genes, memory and psychiatric disorders: focus on c-Fos, Egr1 and Arc. Front. Behav. Neurosci..

[41] Zhang R, Lahens NF, Ballance HI, Hughes ME, Hogenesch JB (2014). A circadian gene expression atlas in mammals: implications for biology and medicine. Proc. Natl Acad. Sci. USA.

[42] Elf J, Li G-W, Xie XS (2007). Probing transcription factor dynamics at the single-molecule level in a living Cell. Science.

[43] Wroblewska A (2018). Protein barcodes enable high-dimensional single-cell CRISPR Screens. Cell.

[44] Tang F (2009). mRNA-Seq whole-transcriptome analysis of a single cell. Nat. Methods.

[45] Hwang B, Lee JH, Bang D (2018). Single-cell RNA sequencing technologies and bioinformatics pipelines. Exp. Mol. Med..

[46] Yamada M, Nagasaki SC, Ozawa T, Imayoshi I (2020). Light-mediated control of gene expression in mammalian cells. Neurosci. Res..

[47] Gossen M, Bujard H (1992). Tight control of gene expression in mammalian cells by tetracycline-responsive promoters. Proc. Natl Acad. Sci. USA.

[48] W W (2017). A light- and calcium-gated transcription factor for imaging and manipulating activated neurons. Nat. Biotechnol..

[49] Lee D, Hyun JH, Jung K, Hannan P, Kwon H-B (2017). A calcium- and light-gated switch to induce gene expression in activated neurons. Nat. Biotechnol..

[50] T K (2013). Functional labeling of neurons and their projections using the synthetic activity-dependent promoter E-SARE. Nat. Methods.

[51] Klapoetke NC (2014). Independent optical excitation of distinct neural populations. Nat. Methods.

[52] Piatkevich KD (2018). A robotic multidimensional directed evolution approach applied to fluorescent voltage reporters article. Nat. Chem. Biol..

[53] Love MI, Huber W, Anders S (2014). Moderated estimation of fold change and dispersion for RNA-seq data with DESeq2. Genome Biol..

[54] Damstra HGJ (2022). Visualizing cellular and tissue ultrastructure using Ten-fold Robust Expansion Microscopy (TREx). eLife.

[55] Sarkar D (2022). Revealing nanostructures in brain tissue via protein decrowding by iterative expansion microscopy. Nat. Biomed. Eng..

