# Supplementary material for: Recording of cellular physiological histories along optically readable self-assembling protein chains
Source: Nat Biotechnol. 2023 Jan 2;41(5):640–51. doi: 10.1038/s41587-022-01586-7 (PMC10188365; doi:10.1038/s41587-022-01586-7)
Supplement: Supplementary file 1 — Supplementary Tables 1 and 2. [file 41587_2022_1586_MOESM1_ESM.pdf]

nature biotechnology

Article

https://doi.org/10.1038/s41587-022-01586-7

# Recording of cellular physiological histories along optically readable self-assembling protein chains

In the format provided by the authors and unedited

# Supplementary information

# Recording of cellular physiological histories along optically readable self-assembling protein chains

Changyang Linghu[1,2,3,4], Bobae An[1,2,3,4], Monika Shpokayte[5,6], Orhan T. Celiker[1,2,3,4], Nava Shmoel[1,2,3,4], Ruihan Zhang[1,2,3,4], Chi Zhang[1,2,3,4], Demian Park[1,2,3,4], Won Min Park[7], Steve Ramirez[6] and Edward S. Boyden*[1,2,3,4,8,9,10,11]

*Email: edboyden@mit.edu

[1]Brain and Cognitive Sciences, MIT, Cambridge, MA 02139

[2]Biological Engineering, MIT, Cambridge, MA 02139

[3]Media Arts and Sciences, MIT, Cambridge, MA 02139

[4]McGovern Institute, MIT, Cambridge, MA 02139

[5]Graduate Program for Neuroscience, Boston University, Boston, MA 02215

[6]Psychological and Brain Sciences, Boston University, Boston, MA 02215

[7]Chemical Engineering, Kansas State University, Manhattan, KS 66506

[8]Howard Hughes Medical Institute, MIT, Cambridge, MA 02139

[9]K Lisa Yang Center for Bionics, MIT, Cambridge, MA 02139

[10]Center for Neurobiological Engineering, MIT, Cambridge, MA 02139

[11]Koch Institute, MIT, Cambridge, MA 02139

## Supplementary Tables

**Supplementary Table 1** - sequences of protein motifs used in this study

| Motif name | Amino acid sequence | Reference |
|---|---|---|
| 1POK (E239Y) | MIDYTAAGFTLLQGAHLYAPEDRGICDVLVANGKIIAVASNIP SDIVPNCTVVDLSGQILCPGFIDQHVHLIGGGGEAGPTTRTPE VALSRLTEAGVTSVVGLLGTDSISRHPESLLAKTRALNEEGIS AWMLTGAYHVPSRTITGSVEKDVAIIDRVIGVKCAISDHRSA APDVYHLANMAAESRVGGLLGGKPGVTVFHMGDSKKALQPI YDLLENCDVPISKLLPTHVNRNVPLFYQALEFARKGGTIDITS SIDEPVAPAEGIARAVQAGIPLARVTLSSDGNGSQPFFDDEGN LTHIGVAGFETLLETVQVLVKDYDFSISDALRPLTSSVAGFLN LTGKGEILPGNDADLLVMTPELRIEQVYARGKLMVKDGKAC VKGTFETA | 1 |
| Maltose binding protein | KIEEGKLVIWINGDKGYNGLAEVGKKFEKDTGIKVTVEHPDK LEEKFPQVAATGDGPDIIFWAHDRFGGYAQSGLLAEITPDKAF QDKLYPFTWDAVRYNGKLIAYPIAVEALSLIYNKDLLPNPPK | 2 |

| | | |
|---|---|---|
| (MBP tag) | TWEEIPALDKELKAKGKSALMFNLQEPYFTWPLIAADGGYAF KYENGKYDIKDVGVDNAGAKAGLTFLVDLIKNKHMNADTD YSIAEAAFNKGETAMTINGPWAWSNIDTSKVNYGVTVLPTFK GQPSKPFVGVLSAGINAASPNKELAKEFLENYLLTDEGLEAV NKDKPLGAVALKSYEEELAKDPRIAATMENAQKGEIMPNIPQ MSAFWYAVRTAVINAASGRQTVDEALKDAQT | |
| HA (HA tag) | YPYDVPDYA | |
| FLAG (FLAG tag) | DYKDDDDK | |
| V5 (V5 tag) | GKPIPNPLLGLDST | |
| 1M3U (D157L, E158L, D161L) | MKPTTISLLQKYKQEKKRFATITAYDYSFAKLFADEGLNVML VGDSLGMTVQGHDSTLPVTVADIAYHTAAVRRGAPNCLLLA DLPFMAYATPEQAFENAATVMRAGANMVKIEGGEWLVETV QMLTERAVPVCGHLGLTPQSVNIFGGYKVQGRGLLAGLQLL SDALALEAAGAQLLVLECVPVELAKRITEALAIPVIGIGAGNV TDGQILVMHDAFGITGGHIPKFAKNFLAETGDIRAAVRQYMA EVESGVYPGEEHSFH | 1 |
| 2CG4 (K126Y, D131Y) | MENYLIDNLDRGILEALMGNARTAYAELAKQFGVSPETIHVR VEKMKQAGIITGARIDVSPKQLGYDVGCFIGIILKSAKDYPSA | 1 |

| | | |
|---|---|---|
| | LAKLESLDEVTEAYYTTGHYSIFIKVMCRSIDALQHVLINYIQ<br>TIYEIQSTETLIVLQNPIMRTIKP | |
| 2VYC<br>(K491L,<br>D494L,<br>D497L) | MKVLIVESEFLHQDTWVGNAVERLADALSQQNVTVIKSTSFD<br>DGFAILSSNEAIDCLMFSYQMEHPDEHQNVRQLIGKLHERQQ<br>NVPVFLLGDREKALAAMDRDLLELVDEFAWILEDTADFIAGR<br>AVAAMTRYRQQLLPPLFSALMKYSDIHEYSWAAPGHQGGVG<br>FTKTPAGRFYHDYYGENLFRTDMGIERTSLGSLLDHTGAFGE<br>SEKYAARVFGADRSWSVVVGTSGSNRTIMQACMTDNDVVV<br>VDRNCHKSIEQGLMLTGAKPVYMVPSRNRYGIIGPIYPQEMQ<br>PETLQKKISESPLTKDKAGQKPSYCVVTNCTYDGVCYNAKEA<br>QDLLEKTSDRLHFDEAWYGYARFNPIYADHYAMRGEPGDHN<br>GPTVFATHSTHKLLNALSQASYIHVREGRGAINFSRFNQAYM<br>MHATTSPLYAICASNDVAVSMMDGNSGLSLTQEVIDEAVDF<br>RQAMARLYKEFTADGSWFFKPWNKEVVTDPQTGLTYLFALA<br>PTKLLTTVQDCWVMHPGESWHGFKDIPDNWSMLDPIKVSIL<br>APGMGEDGELEETGVPAALVTAWLGRHGIVPTRTTDFQIMFL<br>FSMGVTRGKWGTLVNTLCSFKRHYDANTPLAQVMPELVEQ<br>YPDTYANMGIHDLGDTMFAWLKENNPGARLNEAYSGLPVA<br>EVTPREAYNAIVDNNVELVSIENLPGRIAANSVIPYPPGIPMLL<br>SGENFGDKNSPQVSYLRSLQSWDHHFPGFEHETEGTEIIDGIY<br>HVMCVKA | 1 |
| DHF40 | MSSEKEELRERLVKICVELAKLKGDDTLKAAEAAEEAFRLVV<br>LAAMLAGIDSSEVLELAIRLIKTCVVLAAMEGYDISEACRAA | 3 |

| | | |
|---|---|---|
| | AEAFTRVAMAALRAGITSSLVLKAAIELIKECVLNAAVEGYDI SEACRAAAEAFKRVAEAAKRAGITSLETLLRAIEEIRKRVEEA QREGNDISEACRQAAEEFRKKAEELKRRGDV | |
| γPFD | MVNEVIDINEAVRAYIAQIEGLRAEIGRLDATIATLRQSLATL KSLKTLGEGKTVLVPVGSIAQVEMKVEKMDKVVVSVGQNIS AELEYEEALKYIEDEIKKLLTFRLVLEQAIAELYAKIEDLIAEA QQTSEEEKAEEEENEEKAE | 4 |
| Top7 | DIQVQVNIDDNGKNFDYTYTVTTESELQKVLNELKDYIKKQG AKRVRISITARTKKEAEKFAAILIKVFAELGYNDINVTWDGDT VTVEGQLE | 5 |
| dTor_12x 31L | GSSMASGISVEELLKLAKAAYYSGTTVEEAYKLALKLGISVE ELLKLAEAAYYSGTTVEEAYKLALKLGISVEELLKLAKAAYY SGTTVEEAYKLALKLGISVEELLKLAKAAYYSGTTVEEAYKL ALKLGISVEELLKLAEAAYYSGTTVEEAYKLALKLGISVEELL KLAKAAYYSGTTVEEAYKLALKLGISVEELLKLAKAAYYSG TTVEEAYKLALKLGISVEELLKLAEAAYYSGTTVEEAYKLAL KLGISVEELLKLAKAAYYSGTTVEEAYKLALKLGISVEELLKL AKAAYYSGTTVEEAYKLALKLGISVEELLKLAEAAYYSGTTV EEAYKLALKLGISVEELLKLAKAAYYSGTTVEEAYKLALKLG | 6 |
| ERT2-iCre-ERT2 | MAGDMRAANLWPSPLMIKRSKKNSLALSLTADQMVSALLD AEPPILYSEYDPTRPFSEASMMGLLTNLADRELVHMINWAKR VPGFVDLTLHDQVHLLECAWLEILMIGLVWRSMEHPVKLLF APNLLLDRNQGKCVEGMVEIFDMLLATSSRFRMMNLQGEEF | 7 |

| | | |
|---|---|---|
| | VCLKSIILLNSGVYTFLSSTLKSLEEKDHIHRVLDKITDTLIHL MAKAGLTLQQQHQRLAQLLLILSHIRHMSNKGMEHLYSMKC KNVVPLYDLLLEAADAHRLHAPTSRGGASVEETDQSHLATA GSTSSHSLQKYYITGEAEGFPATAVDNLLTVHQNLPALPVDA TSDEVRKNLMDMFRDRQAFSEHTWKMLLSVCRSWAAWCKL NNRKWFPAEPEDVRDYLLYLQARGLAVKTIQQHLGQLNMLH RRSGLPRPSDSNAVSLVMRRIRKENVDAGERAKQALAFERTD FDQVRSLMENSDRCQDIRNLAFLGIAYNTLLRIAEIARIRVKDI SRTDGGRMLIHIGRTKTLVSTAGVEKALSLGVTKLVERWISV SGVADDPNNYLFCRVRKNGVAAPSATSQLSTRALEGIFEATH RLIYGAKDDSGQRYLAWSGHSARVGAARDMARAGVSIPEIM QAGGWTNVNIVMNYIRNLDSETGAMVRLLEDGDLEPSAGD MRAANLWPSPLMIKRSKKNSLALSLTADQMVSALLDAEPPIL YSEYDPTRPFSEASMMGLLTNLADRELVHMINWAKRVPGFV DLTLHDQVHLLECAWLEILMIGLVWRSMEHPVKLLFAPNLLL DRNQGKCVEGMVEIFDMLLATSSRFRMMNLQGEEFVCLKSII LLNSGVYTFLSSTLKSLEEKDHIHRVLDKITDTLIHLMAKAGL TLQQQHQRLAQLLLILSHIRHMSNKGMEHLYSMKCKNVVPL YDLLLEAADAHRLHAPTSRGGASVEETDQSHLATAGSTSSHS LQKYYITGEAEGFPATA | |
| NLS (SV40 NLS) | PKKKRKV | |

| | | |
|---|---|---|
| Linker2 | GG | |
| Linker3 | GSG | |
| Linker4 | GSGG | |
| Linker5 | GGGSG | |
| Linker6 | GGSGGT | |
| Linker7 | GGSGGTG | |
| Linker8 | GGSGGTGG | |
| Linker12 | GGSGGTGGSGGT | |
| Linker13 | GGSGGTGGSGGTG | |
| Linker14 | GGSGGTGGSGGTGG | |
| Linker18 | GGSGGTGGSGGTGGSGGT | |
| Linker24 | GGSGGTGGSGGTGGSGGTGGSGGT | |
| Linker25 | GGSGGTGGSGGTGGSGGTGGSGGTG | |

**Supplementary Table 2** – constructs of self-assembly proteins tested in neurons in this study

| **Construct** (promoters are underlined) | **Resulted pattern of protein self-assembly** (in the cytosol unless noted otherwise) |
|---|---|
| UBC-1POK(E239Y)-Linker25-HA-Linker3-MBP_tag<br>(also known as XRI-HA) | Fiber(s) |

| | |
|---|---|
| UBC-1POK(E239Y)-Linker12-gg-HA | Unstructured aggregates and intertwined fibers |
| UBC-1POK(E239Y)-Linker13-HA-mEGFP | Fiber(s) |
| UBC-1M3U(D157L,E158L,D161L)-Linker14-HA | Unstructured aggregates (and intertwined fibers in a subset of cells) |
| UBC-HA-Linker14-2CG4(K126Y,D131Y) | Uniform expression in the nucleus |
| UBC-2VYC(K491L,D494L,D497L)-Linker14-HA | Nucleus-localized puncta and cytosol-localized puncta |
| CMV-1POK(E239Y)-Linker8-HA | Unstructured aggregates and intertwined fibers |
| UBC-1POK(E239Y)-Linker7-HA-Linker3-MBP_tag | Fiber(s) |
| UBC-HA-Linker3-MBP_tag-Linker18-1POK(E239Y) | Fiber(s) |
| UBC-1POK(E239Y)-Linker5-HA-mEGFP | Fibers (mostly) and puncta |
| UBC-mEGFP-HA-Linker12-1POK(E239Y) | Fiber(s) |
| UBC-1POK(E239Y)-Linker25-HA-g-mEGFP | Fiber(s) |
| UBC-1POK(E239Y)-Linker7-HA-Linker3-Top7 | Short fibers and puncta in the nucleus (mostly) and cytosol |
| UBC-1POK(E239Y)-Linker25-HA-gsg-Top7 | Unstructured aggregates and intertwined fibers in the cytosol; nucleus-localized fibers |

| | |
|---|---|
| UBC-1POK(E239Y)-Linker5-mEGFP-Linker2-HA-Linker3-MBP_tag | Puncta |
| UBC-1POK(E239Y)-Linker24-mEGFP-HA-Linker6-MBP | Fiber(s) with large thickness |
| UBC-1POK(E239Y)-Linker5-mEGFP-HA-Linker3-Top7 | Dense and small puncta |
| UBC-Top7-Linker12-1POK(E239Y)-Linker13-HA-mEGFP | Unstructured aggregates and fibers; high non-assembly background |
| UBC-1POK(E239Y)-Linker24-mEGFP-HA-Linker6-Top7 | Unstructured aggregates and fibers in the cytosol; nucleus-localized fibers |
| UBC-HA-dTor_12x31L-Linker24-1POK(E239Y) | Puncta |
| UBC-NLS-Linker4-1POK(E239Y)-Linker13-HA-mEGFP | Nucleus-localized fiber(s) |
| UBC-NLS-Linker4-1POK(E239Y)-Linker14-HA | Nucleus-localized puncta |
| UBC-DHF40-Linker14-HA | Unstructured aggregates and intertwined fibers |
| UBC-DHF40-Linker13-HA-mEGFP | Unstructured aggregates, puncta, and intertwined fibers |
| UBC-DHF58Four-Linker14-HA | Unstructured aggregates (and intertwined fibers in a subset of cells) in the cytosol and nucleus |

| | |
|---|---|
| UBC-DHF58Six-Linker14-HA | Uniform expression in the nucleus, with dim unstructured aggregates in the cytosol |
| UBC-DHF58Six-Linker14-mRuby2_smFP(HA) | Uniform expression in the nucleus, with dim unstructured aggregates in the cytosol |
| UBC-DHF79-Linker14-HA | Uniform expression in the nucleus, with dim unstructured aggregates in the cytosol |
| UBC-DHF119-Linker14-HA | Uniform expression in the nucleus, with dim unstructured aggregates in the cytosol |
| CMV-DHF40-Linker8-HA | Unstructured aggregates and intertwined fibers |
| CMV-DHF46-Linker8-HA | Puncta |
| CMV-DHF47-Linker8-HA | Unstructured aggregates and puncta |
| CMV-DHF50-Linker8-HA | Unstructured aggregates and puncta |
| CMV-DHF77-Linker8-HA | Unstructured aggregates and puncta |
| UBC-γPFD-Linker8-HA | Puncta |

## References


1. Garcia-Seisdedos, H., Empereur-Mot, C., Elad, N. & Levy, E. D. Proteins evolve on the edge of supramolecular self-assembly. *Nature* **548**, 244 (2017).
2. Kapust, R. B. & Waugh, D. S. Escherichia coli maltose-binding protein is uncommonly effective at promoting the solubility of polypeptides to which it is fused. *Protein Sci.* **8**, 1668–1674 (1999).
3. Shen, H. *et al.* De novo design of self-assembling helical protein filaments. *Science* **362**, 705 (2018).
4. Glover, D. J., Giger, L., Kim, S. S., Naik, R. R. & Clark, D. S. Geometrical assembly of ultrastable protein templates for nanomaterials. *Nat. Commun. 2016 71* **7**, 1–9 (2016).
5. B, K. *et al.* Design of a novel globular protein fold with atomic-level accuracy. *Science* **302**, 1364–1368 (2003).
6. Doyle, L. *et al.* Rational design of α-helical tandem repeat proteins with closed architectures. *Nature* **528**, 585–588 (2015).
7. T, M. & CL, C. Controlled expression of transgenes introduced by in vivo electroporation. *Proc. Natl. Acad. Sci. U. S. A.* **104**, 1027–1032 (2007).
