# Supplementary material for: Recording of cellular physiological histories along optically readable self-assembling protein chains
Source: Nat Biotechnol. 2023 Jan 2;41(5):640–51. doi: 10.1038/s41587-022-01586-7 (PMC10188365; doi:10.1038/s41587-022-01586-7)
Supplement: Supplementary file 2 — Reporting Summary [file 41587_2022_1586_MOESM2_ESM.pdf]

nature research

Corresponding author(s): Edward S. Boyden

Last updated by author(s): Sep 27, 2022

Reporting Summary

Nature Research wishes to improve the reproducibility of the work that we publish. This form provides structure for consistency and transparency in reporting. For further information on Nature Research policies, see our Editorial Policies and the Editorial Policy Checklist.

Statistics

For all statistical analyses, confirm that the following items are present in the figure legend, table legend, main text, or Methods section.

n/a	Confirmed	
☐	☒	The exact sample size (*n*) for each experimental group/condition, given as a discrete number and unit of measurement
☐	☒	A statement on whether measurements were taken from distinct samples or whether the same sample was measured repeatedly
☐	☒	The statistical test(s) used AND whether they are one- or two-sided<br>*Only common tests should be described solely by name; describe more complex techniques in the Methods section.*
☐	☒	A description of all covariates tested
☐	☒	A description of any assumptions or corrections, such as tests of normality and adjustment for multiple comparisons
☐	☒	A full description of the statistical parameters including central tendency (e.g. means) or other basic estimates (e.g. regression coefficient) AND variation (e.g. standard deviation) or associated estimates of uncertainty (e.g. confidence intervals)
☐	☒	For null hypothesis testing, the test statistic (e.g. *F*, *t*, *r*) with confidence intervals, effect sizes, degrees of freedom and *P* value noted<br>*Give P values as exact values whenever suitable.*
☒	☐	For Bayesian analysis, information on the choice of priors and Markov chain Monte Carlo settings
☒	☐	For hierarchical and complex designs, identification of the appropriate level for tests and full reporting of outcomes
☒	☐	Estimates of effect sizes (e.g. Cohen's *d*, Pearson's *r*), indicating how they were calculated

Our web collection on statistics for biologists contains articles on many of the points above.

Software and code

Policy information about availability of computer code

Data collection	Nikon NIS-Elements software was used for image acquisition on fluorescence microscopes.
Data analysis	MATLAB (R2019a), ImageJ (1.53q), Prism (9.4.0), and DESeq2 (1.30.1) in R (4.0.4) were used for data analysis. The custom MATLAB codes for data analysis are available at Zenodo (record 7130256) for public access.

For manuscripts utilizing custom algorithms or software that are central to the research but not yet described in published literature, software must be made available to editors and reviewers. We strongly encourage code deposition in a community repository (e.g. GitHub). See the Nature Research guidelines for submitting code & software for further information.

Data

Policy information about availability of data

All manuscripts must include a data availability statement. This statement should provide the following information, where applicable:

- Accession codes, unique identifiers, or web links for publicly available datasets
- A list of figures that have associated raw data
- A description of any restrictions on data availability

The sequences of XRIs reported in this paper are available at GenBank (accession numbers: OK539810, OK539811, and OK539812). Plasmids generated in this study and their sequences are available at Addgene (plasmid # 178056-178060). The GRCm38 (mm10) reference genome is available at GenBank (accession number: GCA_000001635.2) and the corresponding gene annotations are available at Ensembl (release 88). The data sets generated and analyzed in this study are available at Zenodo (record 7130256) for public access.

Field-specific reporting

Please select the one below that is the best fit for your research. If you are not sure, read the appropriate sections before making your selection.

☒ Life sciences ☐ Behavioural & social sciences ☐ Ecological, evolutionary & environmental sciences

For a reference copy of the document with all sections, see nature.com/documents/nr-reporting-summary-flat.pdf

Life sciences study design

All studies must disclose on these points even when the disclosure is negative.

Sample size	This work describes a new method and does not test a hypothesis, and therefore no sample size estimate was performed. The sample sizes were chosen based on our previous experience in technology development. We found the sample sizes sufficient to yield reproducible results.
Data exclusions	Data were not excluded from analysis.
Replication	Experiments were replicated at least once. All attempts at replication were successful. The detailed experimental protocols are provided to facilitate replication by others.
Randomization	All biological replicates were treated identically, and randomization was not relevant to this study.
Blinding	Samples for all biological replicates were obtained under identical conditions; blinding was not used.

Reporting for specific materials, systems and methods

We require information from authors about some types of materials, experimental systems and methods used in many studies. Here, indicate whether each material, system or method listed is relevant to your study. If you are not sure if a list item applies to your research, read the appropriate section before selecting a response.

Materials & experimental systems

n/a	Involved in the study	
☐	☒	Antibodies
☐	☒	Eukaryotic cell lines
☒	☐	Palaeontology and archaeology
☐	☒	Animals and other organisms
☒	☐	Human research participants
☒	☐	Clinical data
☒	☐	Dual use research of concern

Methods

n/a	Involved in the study	
☒	☐	ChIP-seq
☒	☐	Flow cytometry
☒	☐	MRI-based neuroimaging

Antibodies

Antibodies used	Primary antibodies: anti-HA (Santa Cruz, cat# sc-7392), anti-FLAG (Invitrogen, cat# 740001), anti-V5 (Abcam, cat# ab9113), anti-NeuN (Synaptic Systems, cat# 266004), anti-GFAP (Cell Signaling Technology, cat# 12389), anti-Iba1 (Wako Chemicals, cat# 019-19741), anti-Synaptophysin (Sigma, cat# S5768), anti-Cleaved Caspase-3 (Cell Signaling Technology, cat# 9664), anti-γH2AX (Millipore, cat# 05-636), anti-Hsp70 (Cell Signaling Technology, cat# 4872), anti-Hsp27 (Cell Signaling Technology, cat# 2402). Fluorescent secondary antibodies: Goat anti-Mouse IgG2a Alexa Fluor 647 (Invitrogen, cat# A-21241), Goat anti-Mouse IgG2a Alexa Fluor 546 (Invitrogen, cat# A-21133), Goat anti-Chicken IgY Alexa Fluor Plus 647 (Invitrogen, cat# A-32933), Goat anti-Rabbit IgG Alexa Fluor Plus 647 (Invitrogen, cat# A-32733), Goat anti-Rabbit IgG Alexa Fluor 546 (Invitrogen, cat# A-11035), Goat anti-Guinea Pig IgG Alexa Fluor 488 (Invitrogen, cat# A-11073), Goat anti-Guinea Pig IgG Alexa Fluor 647 (Invitrogen, cat# A-21450), Goat anti-Mouse IgG2a Alexa Fluor 546 (Invitrogen, cat# A-21133), Goat anti-Mouse IgG1 Alexa Fluor 546 (Invitrogen, cat# A-21123), Donkey Anti-Rabbit IgG CF543 (Biotium, cat# 20308).
Validation	Validation statements for use in immunohistochemistry and relevant citations of the primary antibodies used in this study are listed on the manufacturers' website listed below. Citations of the antibodies can also be searched at www.citeab.com<br><br>anti-HA (Santa Cruz, cat# sc-7392): https://www.scbt.com/p/ha-probe-antibody-f-7. Validation included western blot analysis and immunofluorescence staining in HEK293T cells and COS cells transfected with HA-tagged fusion proteins.<br><br>anti-FLAG (Invitrogen, cat# 740001): https://www.thermofisher.com/antibody/product/DYKDDDDK-Tag-Antibody-clone-20H18L16-20H1L23-8H2L5-8H8L17-Recombinant-Polyclonal/740001. Validation included western blot analysis and immunofluorescence staining in HEK293 cells transfected with FLAG-tagged fusion proteins.<br><br>anti-V5 (Abcam, cat# ab9113): https://www.abcam.com/v5-tag-antibody-ab9113.html. Validation included western blot analysis and immunofluorescence staining in HT1080 cells transfected with V5-tagged fusion proteins.

anti-NeuN (Synaptic Systems, cat# 266004): https://sysy.com/product/266004. Validation included immunofluorescence staining in rat neurons and mouse brain slices.

anti-GFAP (Cell Signaling Technology, cat# 12389): https://www.cellsignal.com/products/primary-antibodies/gfap-d1f4q-xp-rabbit-mab/12389. Validation included western blot analysis of extracts from mouse brain, NIH/3T3 cells, rat brain, and C6 cells as well as immunofluorescence staining in rat cerebellum.

anti-Iba1 (Wako Chemicals, cat# 019-19741): https://labchem-wako.fujifilm.com/us/product/detail/W01W0101-1974.html. Validation included immunofluorescence staining in rat cerebral cortex, mouse cerebellum, and mouse retinal whole mount.

anti-Synaptophysin (Sigma, cat# S5768): https://www.sigmaaldrich.com/US/en/product/sigma/s5768. Validation included western blot analysis and immunofluorescence staining in rat cerebellum sections.

anti-Cleaved Caspase-3 (Cell Signaling Technology, cat# 9664): https://www.cellsignal.com/products/primary-antibodies/cleaved-caspase-3-asp175-5a1e-rabbit-mab/9664. Validation included western blot analysis of extracts from C6 (rat), NIH/3T3 (mouse), and Jurkat (human) cells, untreated or treated with staurosporine or etoposide, and immunofluorescence staining in mouse embryo (in the presence of control peptide or Cleaved Caspase-3 (Asp175) blocking peptide), Jurkat cells (untreated or treated with etoposide), and HT-29 cells (untreated or treated with staurosporine).

anti-γH2AX (Millipore, cat# 05-636): https://www.sigmaaldrich.com/US/en/product/mm/05636. Validation included western blot analysis of Jurkat cell lysates untreated or treated with staurosporine and immunofluorescence staining of Jurkat cells treated with etoposide.

anti-Hsp70 (Cell Signaling Technology, cat# 4872): https://www.cellsignal.com/products/primary-antibodies/hsp70-antibody/4872. Validation included western blot analysis of extracts from HeLa, NIH/3T3, C6 and COS cells as well as immunohistochemical analysis of paraffin-embedded human breast carcinoma, colon carcinoma, lung carcinoma, Non-Hodgkin's lymphoma, and prostate carcinoma.

anti-Hsp27 (Cell Signaling Technology, cat# 2402): https://www.cellsignal.com/products/primary-antibodies/hsp27-g31-mouse-mab/2402. Validation included western blot analysis of extracts from COS-7 cells, HSP27 knock-out cells, HeLa cells, and HeLa cells transfected with control siRNA or HSP27 siRNA as well as Immunohistochemical analysis of A549 cells and paraffin-embedded human lung carcinoma, breast carcinoma untreated or treated with lambda phosphatase.

Eukaryotic cell lines

Policy information about cell lines

Cell line source(s)	U2OS cell line (human bone osteosarcoma epithelial cells) from ATCC.
Authentication	The cell line was authenticated by the manufacturer via STR profiling.
Mycoplasma contamination	The cell line was tested for mycoplasma contamination by the manufacturer to their standard levels of stringency (mycoplasma contamination was not detected).
Commonly misidentified lines (See ICLAC register)	The U2OS cell line was used here because it is a common cell line for testing new tools.

Animals and other organisms

Policy information about studies involving animals; ARRIVE guidelines recommended for reporting animal research

Laboratory animals	Male and female Swiss Webster mice at postnatal day 0 or 1 (Taconic). Male wild type C57BL/6 mice at 3 months of age (Charles River Labs).
Wild animals	The study did not involve wild animals.
Field-collected samples	The study did not involve field-collected samples.
Ethics oversight	All procedures involving animals at the Massachusetts Institute of Technology were conducted in accordance with the US National Institutes of Health Guide for the Care and Use of Laboratory Animals and approved by the Massachusetts Institute of Technology Institutional Animal Care and Use and Biosafety Committees. All procedures involving animals at Boston University were conducted in accordance with the US National Institutes of Health Guide for the Care and Use of Laboratory Animals and approved by the Boston University Institutional Animal Care and Use and Biosafety Committees.

Note that full information on the approval of the study protocol must also be provided in the manuscript.
